# Natural polyphenols as sirtuin 6 modulators

**DOI:** 10.1038/s41598-018-22388-5

**Published:** 2018-03-07

**Authors:** Minna Rahnasto-Rilla, Jonna Tyni, Marjo Huovinen, Elina Jarho, Tomasz Kulikowicz, Sarangan Ravichandran, Vilhelm A. Bohr, Luigi Ferrucci, Maija Lahtela-Kakkonen, Ruin Moaddel

**Affiliations:** 1 Biomedical Research Center, National Institute on Aging, National Institutes of Health, Baltimore, Maryland 21224 USA; 2 School of Pharmacy, University of Eastern Finland, P.O.Box 1627, 70210 Kuopio, Finland; 3 Advanced Biomedical Computing Center, Frederick National Laboratory for Cancer Research, Leidos Biomedical Research Inc, Fredrick, Maryland 21701 USA

## Abstract

Flavonoids are polyphenolic secondary metabolites synthesized by plants and fungus with various pharmacological effects. Due to their plethora of biological activities, they have been studied extensively in drug development. They have been shown to modulate the activity of a NAD^+^-dependent histone deacetylase, SIRT6. Because SIRT6 has been implicated in longevity, metabolism, DNA-repair, and inflammatory response reduction, it is an interesting target in inflammatory and metabolic diseases as well as in cancer. Here we show, that flavonoids can alter SIRT6 activity in a structure dependent manner. Catechin derivatives with galloyl moiety displayed significant inhibition potency against SIRT6 at 10 *µ*M concentration. The most potent SIRT6 activator, cyanidin, belonged to anthocyanidins, and produced a 55-fold increase in SIRT6 activity compared to the 3–10 fold increase for the others. Cyanidin also significantly increased SIRT6 expression in Caco-2 cells. Results from the docking studies indicated possible binding sites for the inhibitors and activators. Inhibitors likely bind in a manner that could disturb NAD^+^ binding. The putative activator binding site was found next to a loop near the acetylated peptide substrate binding site. In some cases, the activators changed the conformation of this loop suggesting that it may play a role in SIRT6 activation.

## Introduction

Flavonoids are a large family of naturally occurring polyphenolic compounds that provide important health benefits and help to protect against cancer, cognitive decline, diabetes, heart disease, and obesity.

**Figure 1:**
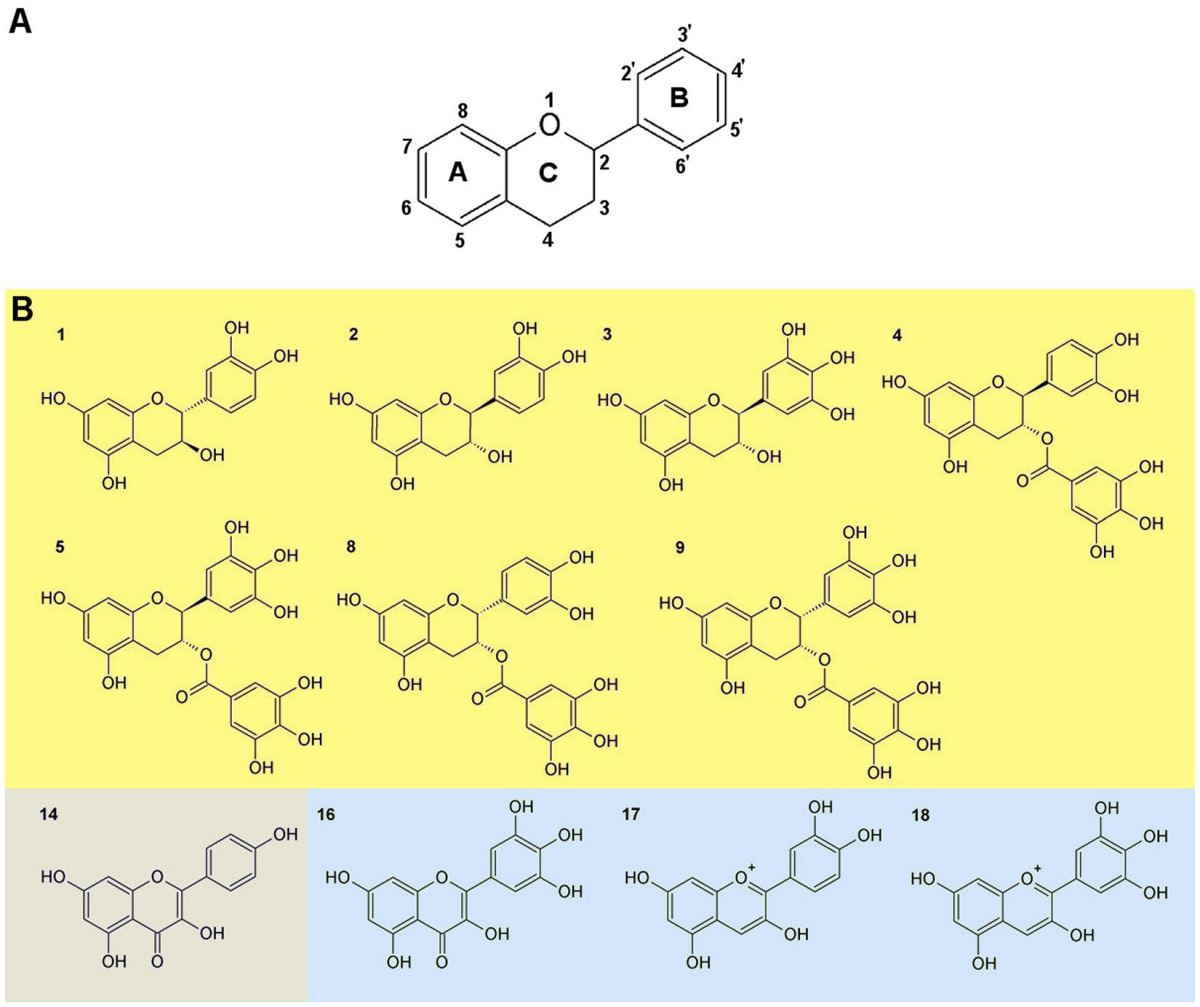
General scaffold of flavonoids (**A**). The structures of the most potent SIRT6 modulators are displayed, and additional structures are available in supplementary Tables S1 and S2. The yellow background represents inhibitors, gray represents inhibitors and activators and blue represents activators (**B**).

Chemically flavonoids contain a fifteen-carbon skeleton consisting of two benzene rings (A and B) linked via a heterocyclic pyran ring (C) (Fig. 1A). They are synthesized via the phenylpropanoid pathway, representing a rich source of metabolites in plants. The chemical nature of flavonoids depends on their structural class, degree of hydroxylation, other substitutions and conjugations, and the degree of polymerization. Flavonoids are classified in five major structural classes including flavan-3-ols, flavanones, flavones, flavonols, and anthocyanidins. Catechins share a general flavan-3-ol structure whereas flavanones, flavones and flavonols include a carbonyl group on position 4. Flavones, flavonols and anthocyanidins also contain a double bond between positions 2 and 3.

The antioxidant property of flavonoids may be mediated by many mechanisms including inhibition of enzymes involved in free radical generation and subsequently suppression of reactive oxygen species (ROS). Sirtuins (SIRTs) is an enzyme family that can modulate ROS levels notably during calorie restriction, which has been shown to enhance lifespan for several organisms. SIRTs are nicotinamide adenine dinucleotide (NAD^+^) dependent histone deacetylases that catalyze the removal of acetyl group from lysine residue. Among the seven-membered mammalian sirtuin family, SIRT6 deacetylates histone 3 lysine 9 (H3K9)^1,2^ and 56 (H3K56)^3^ and also displays mono-ADP ribosyltransferase^4^ and deacylase activities^5^. These functions of SIRT6 are involved in the regulation of many genes including stress responses. SIRT6 deficient cells display sensitivity to oxidative stress and a reduced capacity for DNA repair^6,7^ and SIRT6 knockout mice show many hallmarks of premature aging. Adversely, male mice overexpressing SIRT6 have a significantly longer lifespan than their wild-type counterparts^8,9^. SIRT6 also plays an important role in controlling glucose and lipid metabolism, regulating the expression of multiple glycolytic and lipid genes involved in cellular response^10,11^. These diverse functions of SIRT6, highlights its importance in aging and protecting many cellular functions. Therefore, compounds that can regulate SIRT6 activities are considered as promising therapeutics for age-related diseases including cancer, diabetes, neurodegenerative diseases and metabolic disorders.

Resveratrol is a widely studied polyphenolic^12^ antioxidant and is known to increase the deacetylation activity of SIRT1 through a mechanism that is still not fully understood. So far only a few compounds have been identified to regulate the deacetylation activity of SIRT6. For example, long-chain free fatty acids stimulated deacetylation^13^ activity, and polyphenols, specifically quercetin and luteolin, were shown to increase SIRT6 deacetylation activity at high concentrations, while also inhibiting deacetylation activity at low concentrations^14^. In addition, the known sirtuin inhibitor EX-527^15^ and a group of peptides and pseudopeptides^16^ were reported as SIRT6 inhibitors, but they did not exhibit selectivity towards SIRT6. Quinazolinedione derivatives, were also recently discovered^17,18^ to inhibit SIRT6 deacetylation activity. To the best of our knowledge, this is the first work that shows rational structure activity relationship (SAR) for SIRT6 to compare inhibition and activation of SIRT6 deacetylation activity using chemically diverse compounds. The present study evaluated the differences in chemical features between SIRT6 inhibitors and SIRT6 activators. Molecular docking was also carried out to discover their binding sites on SIRT6, and to identify major interactions occurring on the enzyme active site with inhibitors and activators. This represents an expansion of the chemical spectrum of SIRT6 modulators. Here we showed that anthocyanidins strongly increase SIRT6 deacetylation activity *in vitro*. Moreover, the most potent activator, cyanidin, up-regulated SIRT6 protein expression on the human colon adenocarcinoma Caco-2 cells.

## Results

### Flavonoids modulate SIRT6 deacetylation activity *in vitro*

**Figure 2:**
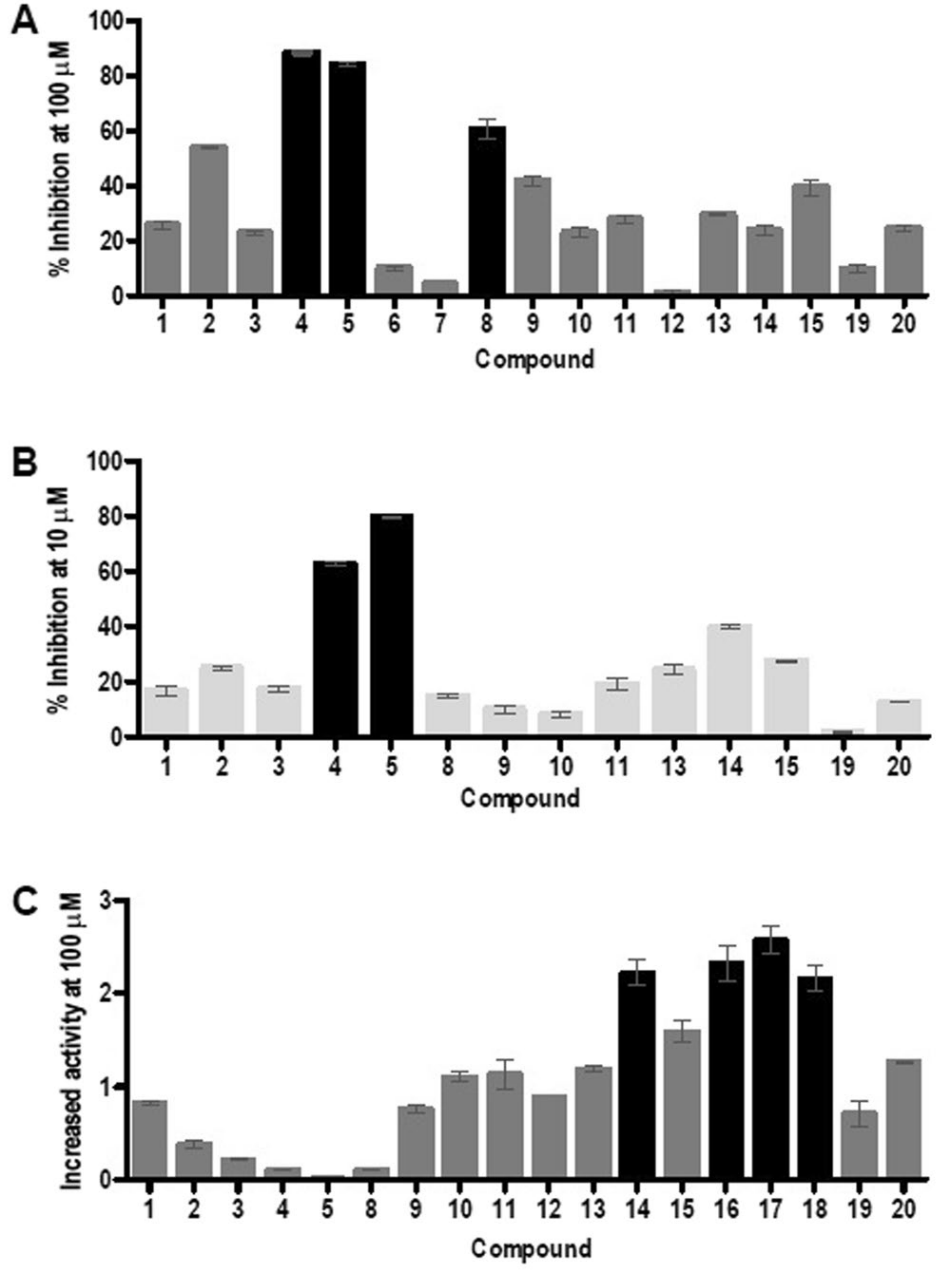
SIRT6 modulation by selected flavonoids. Inhibition % at the 100 *µ*M concentration (**A**), at the 10 *µ*M concentration (**B**) of flavonoids. Black bars indicate ≥60% inhibition. Activation in the presence of 100 *µ*M flavonoids (**C**). Black bars represent more than 2 -fold activation. The data is presented as mean ± SD, (n = 3).

A set of flavonoids (Fig. 1B; Table S1) and phenolic acids (Table S2) were tested using recently developed HPLC-based SIRT6 assay^14,19,20^ with two substrate concentrations by determining the level of deacetylated peptide H3K9. SIRT6 activity was determined at multiple concentrations of the tested compounds (Fig. 2), to determine whether they modulated (increased/decreased) SIRT6 deacetylation activity. The developed assay was carried out in the presence of GST-tagged SIRT6, NAD^+^ and H3K9Ac with tested polyphenols.

The observed deacetylation activity of flavonoids is shown in Fig. 2. Of the tested compounds, catechins showed inhibition of the deacetylation activity of SIRT6, whereas anthocyanidins (Fig. 2C) increased the deacetylation activity. Flavonoids can inhibit or activate deacetylase activity of SIRT6 depending on the concentration. The inhibition of compounds **4** and **5** was significant at a concentration of 10 *µ*M (Table 1; Suppl. Fig. S1), while compounds **16**–**18** more than doubled SIRT6 deacetylase activity. Their respective *IC*_50_ and *EC*_50_ were determined (Table 1; Supp. Fig. S1). Maximal activation was determined at saturating concentrations of the tested compounds and indicates the maximal effect of the tested compounds in the *in vitro* assay.

**Table 1:** Dose response data of modulators. Data are presented as mean ± SD, (n = 3).

	Compound	*IC*_50_ value (*µ*M)	
4	(−)-Catechin gallate	2.5 ± 0.03	
5	(−)-Gallocatechin gallate	5.4 ± 0.04	
	**Compound**	***EC***_**50**_ **value** **(** ***µ*** **M)**	**Maximal activation (fold)**
13	Luteolin^14^	270 ± 25	6.1
14	Kaempferol	n.d	3.0
15	Quercetin^14^	990 ± 250	10
16	Myricetin	404 ± 20	7.7
17	Cyanidin	460 ± 20	55
18	Delphinidin	760 ± 200	6.3

#### Catechins

Catechins and epicatechins are stereoisomers that results in different inhibition towards SIRT6. Catechins with trans configuration (catechins) are more potent SIRT6 inhibitors than cis stereoisomeric analogs (epicatechins). (+)-Enantiomer (**1**) showed only 26% inhibition at 100 *µ*M concentration towards SIRT6 but for (−)-enantiomer (**2**) the inhibition was 54%. (−)-Gallocatechin was a weak inhibitor, similar to compound **1**. The galloyl moiety on the carbon 3 seems to significantly increase deacetylation activity of SIRT6. This was also observed in case of compounds **4**, **5** and **8** in which inhibition exceeded 60% whereas compounds **6** and **7** showed less than 10% inhibition towards SIRT6. Compound **9** showed moderate inhibition towards SIRT6. The most potent inhibitors, **4** and **5** displayed *IC*_50_ values of 2.5 *µ*M and 5.4 *µ*M, respectively (Table 1).

#### Flavanones

The carbonyl group at position 4 in compounds **10** and **11** did not improve the inhibitory activity towards SIRT6 compared to compound **1**. O-Glycosylation occurs primarily on position 5 and 7 on the A ring and, C-glycosylation primarily occurs on position 6 and 8 on the A-ring. Glycosylation by glucose on these carbons displayed weaker inhibition activity compared to the compounds with basic flavonoid scaffold (data not shown).

#### Flavones and flavonols

The hydroxyl group on the carbon 3 improved the inhibition potency of compound **13** compared with **12**. Compound **13** was a weak inhibitor and it increased deacetylation with maximal activation of 6-fold although at higher concentrations. Both flavonols **14** and **15** were inhibitors as well as activators depending on the concentration of compound. Surprisingly, compound **16** with the hydroxyl group on the position 5′ (*R*_3_) increased SIRT6 activity.

#### Anthocyanidins

Two anthocyanidins (**17** and **18**) were tested and both showed an increase in deacetylase activity for SIRT6. Compound **17** was significantly more effective producing 55-fold maximal activation (Table 1) compared to the other activators with maximal activation of 3–10 -fold. In general, *EC*_50_ values of activators varied from 270 *µ*M of compound **13** to 990 *µ*M of compound **15**. Compound **18** with three hydroxyl groups (*R*_1_, *R*_2_ and *R*_3_) displayed weaker activity against SIRT6 than compound **17**. The *in vitro* SIRT6 deacetylation activity for cyanidin (**17**) and delphinidin (**19**) was also determined by western blot analysis using the core histones and determining the remaining levels of histone H3 acetylated on lysine 9 (Suppl. Fig. S2). Both compounds increased deacetylation activity ∼2.5 fold at 100 µM.

#### Isoflavones

These compounds are structurally similar to estrogens and are also known as phytoestrogens. Two isoflavones (**19** and **20**) were tested and both were weak SIRT6 inhibitors but compound **20** was also able to activate deacetylation of SIRT6. The methoxy moiety seems to improve slightly the inhibition potency toward SIRT6 although the result was ambiguous, since the inhibition potency of compound **20** was same level as compounds **10** and **14**.

#### Phenolic acids

A set of phenolic acids (gallic acid derivatives), which is another main class of plant polyphenols, were also included in the study. Although compounds **22** and **23** increased slightly SIRT6 activation, overall phenolic acids (**22**–**27**) were weaker modulators than flavonoids.

### Cyanidin up-regulates SIRT6 and FoxO3α protein expression and downregulates Twist1 and GLUT1 expression in Caco-2 cells

**Figure 3:**
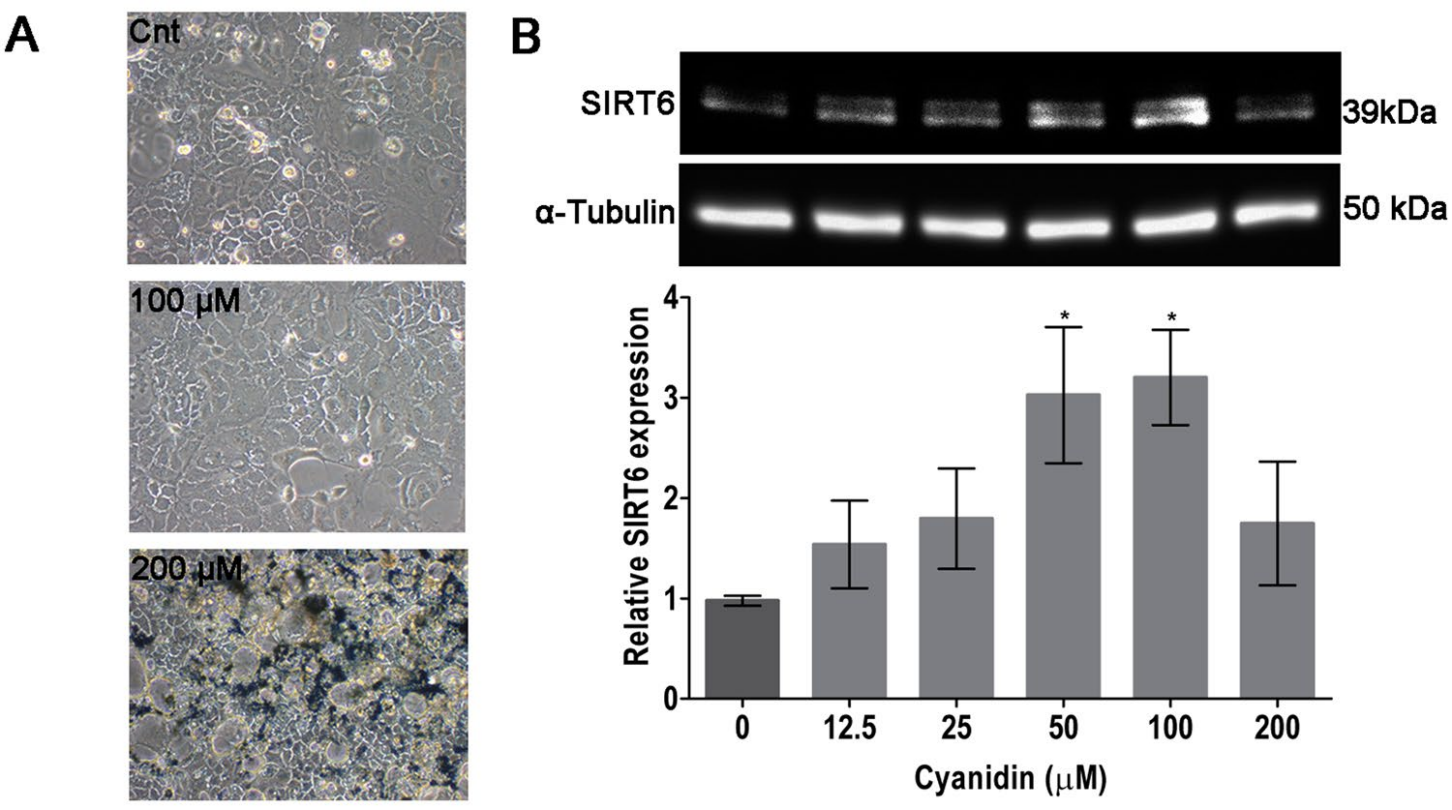
Caco-2 cells after cyanidin treatment and expression of SIRT6 protein. Cells were exposed to 0.5% DMSO (control) or various concentration of compound **17** (n = 5) for 24 h. (**A**) Representative light microscopy images of Caco-2 cells after control (Cnt), 100 *µ*M cyanidin or 200 *µ*M cyanidin treatment. (**B**) Immunoblotting analysis of SIRT6 protein. SIRT6 protein levels (MW: 39 kDa) were normalized relative to *α*-tubulin (MW: 50 kDa) and quantification is represented as fold change respect to control. Values are expressed as mean ± SEM of four independent experiment (*p values < 0.05; one way-ANOVA). SIRT6 expression were determined by immunoblotting (**B**).

**Figure 4:**
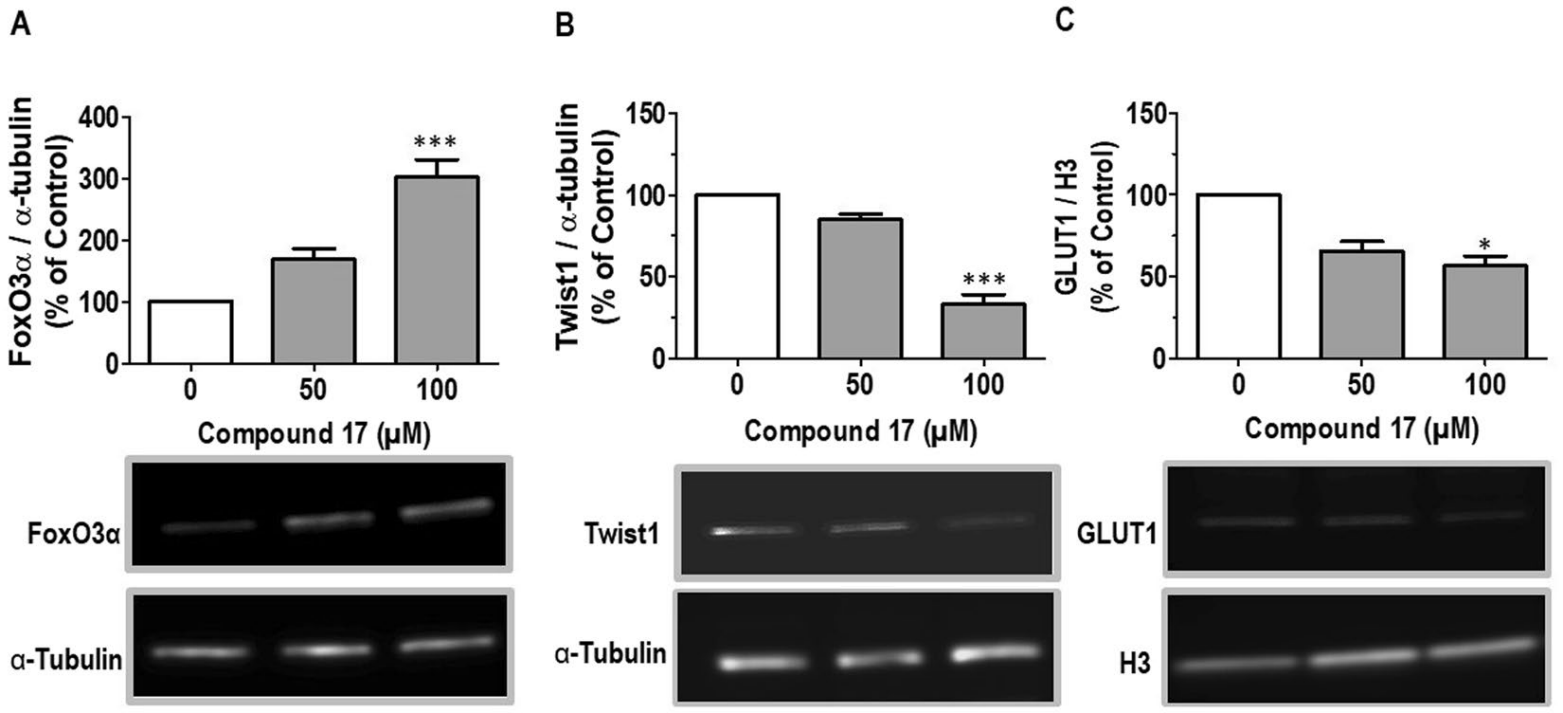
Cyanidin (compound **17**) effect on the expression of FoxO3α (**A**), Twist1 (**B**) and GLUT1 (**C**) protein. Caco-2 cells were treated with DMSO (white bar) or 50 and 100 µM compound **17** (grey bars) for 24 h. FoxO3α, Twist1 and GLUT1 expression were quantified and normalised with α-tubulin or H3. Data represent the mean ± SEM of three independent experiments,*p < 0.05, **p < 0.01 and ***p < 0.001 between the indicated groups.

In order to assess the effects of the most potent activator on SIRT6 expression, Caco-2 cells at passages 30–40 were exposed to DMSO (control) or various concentrations of compound **17** (12.5–200 *µ*M) for 24 h. After the treatment, conditions of the cells were evaluated under a light microscope (Fig. 3A). Cells treated with 12.5 *µ*M to 100 *µ*M of compound **17** were similar to control cells, whereas at 200 *µ*M compound **17** precipitated out of solution. Immunoblotting analysis of total Caco-2 protein lysates (Fig. 3B, Suppl. Fig. S3) demonstrated that compound **17** was effective in a dose-dependent manner after 24 hour exposure, while cells treated by 50–100 *µ*M concentration showed significantly increased SIRT6 expression with 3.5 fold up-regulation. In addition, the effect of compound **17** on the expression levels of the transcription factor forkhead box O-3α (FoxO3α), Twist-related protein 1 (Twist1) and glucose transporter (GLUT1) were studied at 50–200 µM concentrations, and subsequently Caco2 lysates were analyzed by immunoblotting. Compound **17** enhanced the protein expression of FOXO3 significantly (Fig. 4A), but downregulated Twist1 (Fig. 4B) and GLUT1 (Fig. 4C) expression at 100 µM concentration. Full-length blots are presented in Supplementary Fig. S4.

### The binding sites of flavonoids in SIRT6

**Figure 5:**
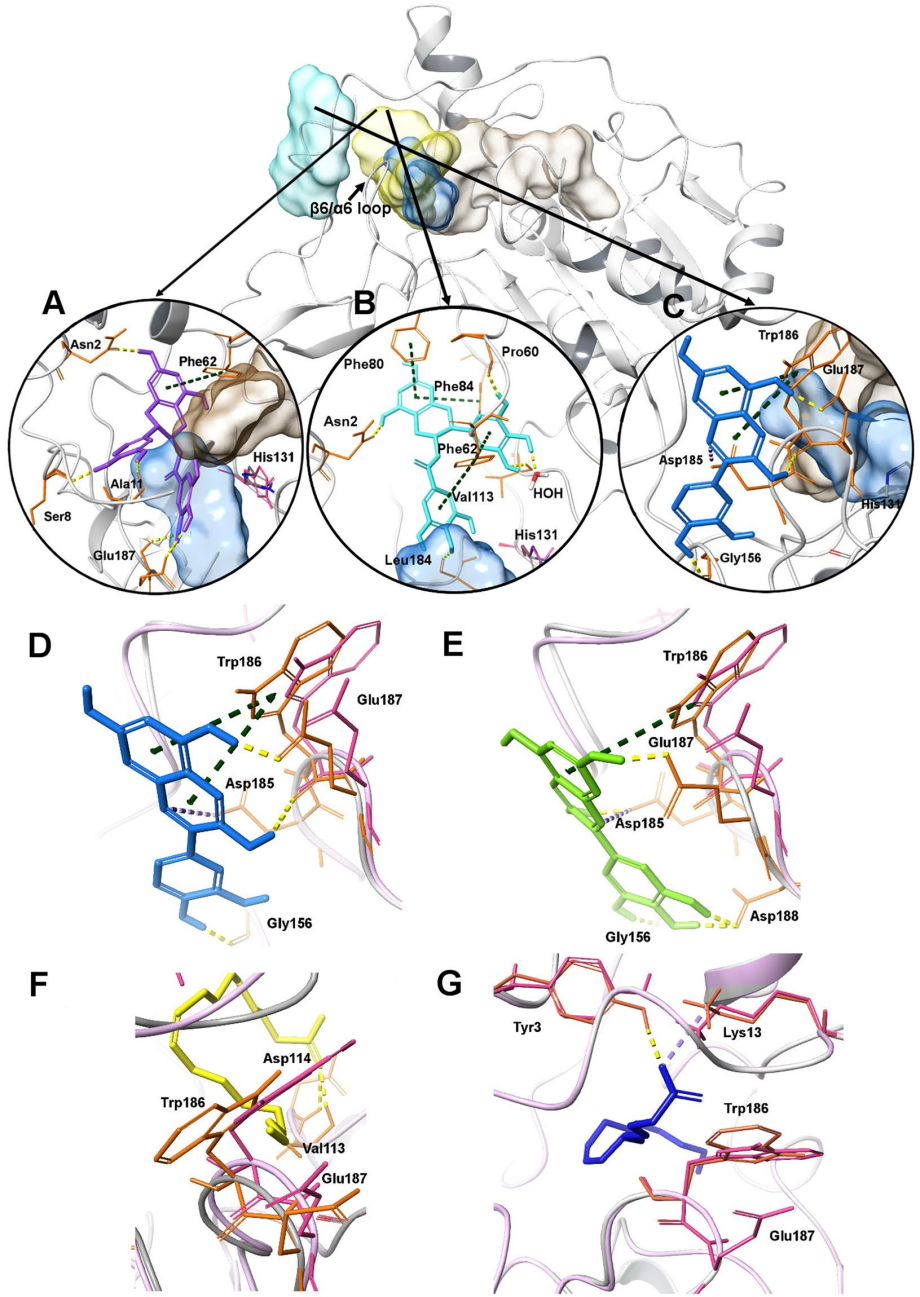
SIRT6 and locations of binding sites of activators (light turquoise), inhibitors (yellow), peptide substrates (blue) and for NAD^+^ (brownish gray). Close-up view of the interactions of best inhibitors, compound **4** (**A**) and compound **5** (**B**) and best activator, compound **17** (**C**). The best activators compound **17** (**D**) and compound **18** (**E**) induce changes on the β6/α6 loop and the orientation of Trp186 and Glu187 similar to known activators oleic acid (**F**) and linoleic acid (**G**). Interactions: Yellow dashes indicate hydrogen-bonding, dark green dashes indicate *π*-*π* stacking and light purple dash indicates salt bridge (interaction to Asp185). Pink residues and loops indicate the original residue and loop orientation in the protein structure before inhibitor or activator binding.

To study the interactions, molecular docking studies of flavonoids were performed in the binding sites of the human SIRT6 (PDB entry:3ZG6, resolution 2.2 Å)^5^. The research showed that the inhibitors bound quite close to the binding site of nicotinamide (NAM) moiety of NAD^+^. The most potent inhibitor, compound **4** (Fig. 5A) occupied partially the peptide substrate binding site and subsequently prevented the active histidine (His131) to orient towards NAD^+^ for reaction. Compound **4** formed interactions with residues Asn2, Ser8, Ala11, Phe62, and Glu187. However, most commonly the binding pose of inhibitors corresponded to the pose of compound **5** (Fig. 5B). This site resembles the binding site of co-crystallized inhibitor Ex-527 in SIRT1.

The activity of SIRT6 inhibitors was increased *in vitro* when the hydroxyl group at position 3 (Fig. 1A) was replaced by a galloyl moiety. The overall comparison of compounds **3** and **5** revealed that compound **5** can occupy a larger volume of the inhibitor binding pocket than compound **3** (Suppl. Fig. S5). A closer investigation showed that compound **5** can form additional interactions within the binding site involving the following residues: Pro60, Phe62, Phe80, Phe84 and Leu184 (Suppl. Fig. S6). The pose comparison of compounds **6** and **8** also showed the importance of galloyl moiety, as it ensured the interaction to Leu184 which is located deep in the pocket while the other moieties of compound **6** could interact with other parts of the inhibitor binding pocket (Suppl. Fig. S7). A comparison of compounds **3** and **7** together with compounds **5** and **9** was carried out to examine how the configuration of the galloyl moiety affected the inhibition potency. Compound **7** did not reach as deep into the binding pocket as did compound **3** (Suppl. Fig. S8). Although the position of compounds **9** and **5** were similar in the inhibitor pocket, compound **5** formed more interactions with residues Phe62 and Phe84 (Suppl. Fig. S9).

The additional carbonyl group (ring C; Fig. 1A) in compounds **10** and **11** did not result in additional interactions when compared to compound **1**. Although there was no major difference in the binding poses of compounds **12** and **13** at the inhibitor binding site, compound **13** could form more interactions than compound **12** in majority of the poses. Interestingly, the methoxy moiety in compound **20** did not contribute any additional interactions in the docking studies compared to compound **19**. Phenolic acids (compounds **22–27**), on the other hand, occupied only a limited volume of the inhibitor binding pocket (Suppl. Fig. S10), resulting in decreased interactions, which may explain their poor inhibitory potency. Compounds binding to the putative inhibitor/activator binding sites using 2D interaction diagrams are presented in Supplementary Figures S11–S16 and S17–S20, respectively.

The activator binding site was discovered with SiteMap. SiteMap uses different scoring functions to assess the found sites. One of these functions is SiteScore, which evaluates if the site is likely to bind a drug or not. Scores over 1.0 are defined to be promising drug-binding sites, and sites having scores under 0.8 most likely will not bind drugs. The putative activator site had a SiteScore of 1.003, and was located close to the β6/α6 loop region (Fig. 5). All activators formed interactions at the β6/α6 loop region with Trp186 and/or Glu187. Some of the activators had additional interactions with Gly156, Asp185 and Asp188. The most potent activator, compound **17** (Fig. 5C) formed all of these interactions except for the interaction with Asp188. Unlike the other activators, compounds **16**, **17** and **18** interacted with Asp185 at the activator binding site, which may be responsible for their increased activity (Suppl. Fig. S21).

Some of the activators changed the orientation of Trp186 and/or Glu187 and some altered the conformation of β6/α6 loop. Two of these were the most potent activators, compounds **17** and **18** (Fig. 5E). Similarly, the docking of oleic acid (Fig. 5F) and linoleic acid (Fig. 5G), known SIRT6 activators, also resulted in changes in the Trp186 and/or Glu187 orientation and/or β6/α6 loop conformation. These results suggest that changes in the Trp186 and/or Glu187 orientation and β6/α6 loop conformation could be factors that are involved in the activation of SIRT6. Subsequently, an impact analysis of the key activator residues were carried out (Suppl. Tables S3 and S4). The results demonstrate that only residue Asp188 is not conserved and can accommodate a wide range of substitutions (Suppl. Table S3).

Dual modulators, such as compound **14**, could form interactions with the same amino acid residues that interacted with the most potent activator/inhibitors with their respective binding sites. Compound **14** had similar interactions with Pro60, Phe62, Val113 and Leu184 as did the potent inhibitor, compound **5**, at the inhibitor site (Suppl. Fig. S22). It also had interactions with, Glu187 and Asp188, as did the most potent activators, compounds **17** and **18**, at the putative activator site. Compound **14** also induced a minor change on the conformation of β6/α6 loop, but it did not change the orientation of Trp186, nor did it form an interaction with Trp186 (Suppl. Fig. S22).

## Discussion

Among sirtuins, SIRT6, has been implicated in aging and age-related diseases, but its physiological role is not completely understood. The extent to which increased SIRT6 activation affects these disease conditions is still unclear; it might offer a protective mechanism or, alternatively, represent part of the disorder process. Although there is considerable evidence that SIRT6 is a tumor suppressor, the effect is double-edged since it can also inactivate tumor suppressor proteins FoxO3a and p53. To further examine these opposite roles of SIRT6, there is a definite need for novel potent SIRT6 modulators, for both inhibitors and activators. These modulators make it possible to study the physiological role and therapeutic potential of SIRT6.

Several SIRT6 inhibitors have been reported previously^16,17^ and the most potent compounds have been discovered against the acylation activity of SIRT6^21,22^. SIRT6 preferentially removes long chain fatty acyl lysine *in vitro* compared to the deacetylation of target substrates^5^. SIRT6 has been shown to efficiently deacetylate lysines 9 (H3K9) and 56 (H3K56) on the H3 sequence *in vivo*^2,3^. H3K9 is the specific regulation site of chromatin at telomeres^1,23,24^ while the acetylation status of H3K56 controls DNA damage response and genomic stability^3,25^.

In the present study, catechins exhibited inhibition activity against SIRT6 catalyzed H3K9Ac deacetylation. Catechins are a major component of green tea and in recent years many health benefits associated with the consumption of green tea have been reported. Green tea has been suggested to reduce ROS production and subsequently exhibit protective role against oxidative stress mediated diseases. Interestingly, catechins have also been demonstrated to protect cells against oxidative stress and DNA damage by increasing the activity of SIRTs. Tao *et al*. 2015 reported that (-)-epigallocatechin-3-gallate induced oxidative stress in cancer cells but it had protective role in normal cells, which was linked to the increased SIRT3 activity^26^. In addition, (-)-epigallocatechin-3-gallate has been reported to extend the lifespan in rats, which was consequence of activation of SIRT1 and protection against oxidative stress^27^. More studies are needed to reveal the role (−)-epigallocatechin-3-gallate may play with sirtuins in oxidative stress.

Interestingly, the most prominent activators for SIRT6 among the flavonoids were the anthocyanidins, the universal plant pigment, responsible for the red, purple, and blue color in many fruits, vegetables and flowers. The most potent compound in the class of anthocyanidins, cyanidin, significantly increased the deacetylation activity of SIRT6. It is most abundant in red berries including bilberry, raspberry and cranberry. Studies have suggested that anthocyanidins, including cyanidin, may play important roles in helping to reduce the risk of many age-related diseases. The effect has been linked to their protective effect against oxidative stress, which results in the decreased production of ROS and nitrogen species^28–30^. Cell culture and *in vivo* studies of anthocyanidins and their glycosylated counterparts (anthocyanins) revealed anticarcinogenic properties against colon, skin, and lung cancer. While laboratory studies have provided some insight into how anthocyanins may work, the exact mechanism for how these compounds prevent cancer is unclear. Thus far studies in a variety of cancer cells revealed that anthocyanins activate detoxifying enzymes, prevent cancer cell proliferation, induce cancer cell apoptosis and have anti-inflammatory and antiangiogenic effects^31,32^. To the best of our knowledge, this is the first study that showed the up-regulation of SIRT6 in colon adenocarcinoma Caco-2 cells treated by cyanidin.

Additionally, cyanidin affected the expression levels of SIRT6 associated genes such as FoxO3α, Twist1 and GLUT1. FoxO3α belongs to the family of forkhead box transcription factors that play important roles in regulating the expression of genes involved in cell growth, proliferation, differentiation, and longevity. Deregulation of FoxO3 is involved in tumorigenesis. Previous studies reported that FoxO3α gene is regulated by SIRT6 which forms a complex with FoxO3α in the nucleus, and further induces the expression of genes involved in antioxidation^33^. Embryonic transcription factors Twist1 and glucose transporter GLUT1 are overexpressed in many tumors. SIRT6 suppress cell proliferation via Twist1, which is also a key factor in the promotion of metastasis of cancer cells^34^. SIRT6 regulates the expression of many glycolytic genes via the hypoxia inducible factor-1 (HIF1)-alpha pathway^10^. SIRT6 was recently shown to regulate metabolic reprogramming in cancer cells via metabolic signaling pathways decreasing the expression of glycolytic genes, including GLUT1 and HIF1-alpha and decreasing glucose uptake and lactate formation by cells.

Sirtuins are involved in a number of central physiological processes, and their activity are likely regulated by endogenous signaling pathways in a tissue-specific and signal-dependent manner at various levels. Sirtuins can be regulated by many mechanisms, including transcriptionally and post-translationally by changing the stability, activity, localization or degradation of the protein. In addition, protein complex formation with other binding partner proteins and changes in NAD^+^ availability may play an important role in regulation of sirtuin functions and expression. Many enzymes such as nicotinamide phosphoribosyltransferase (NAMPT) are involved in NAD^+^ synthesis, which is upregulated by AMP-activated protein kinase (AMPK)^35^. Polyphenols such as resveratrol^36^ and anthocyanins^37^ have been reported to activate AMPK, an effect that may be mediated by SIRT1. Thus, the activation of AMPK by polyphenols such as cyanidin may be one possible mechanism to upregulate SIRT6 expression in Caco-2-cells. Alternatively, recently it has been demonstrated that activation of PPARγ via rosiglitazone increases SIRT6 expression^38^. Moreover, anthocyanins have been shown to induce PPARγ expression^39^, and in a separate study it was demonstrated that anthocyanin-rich berries increased PPARγ activity as well^40^. These studies demonstrate that the observed increase in SIRT6 expression may result from changes in multiple pathways.

Molecular docking studies were carried out to identify interactions occurring between diverse flavonoid classes and SIRT6.The results revealed existence of diverse possible binding sites for inhibitors (yellow region) and activators (turquoise region) (Fig. 5). The binding site of most of the inhibitors was situated close to the binding site of known sirtuin inhibitors, Ex-527 and NAM^41,42^. The site was located at highly conserved region at the cleft between two domains, a large Rossman fold domain and a smaller zinc binding domain^41^. The cleft also forms the pocket for the acetylated sirtuin substrate (blue region Fig. 5) and for the co-factor, NAD^+^ (brownish gray region). The most potent inhibitor, compound **4**, could partially occupy the binding site of the acetylated sirtuin substrate. Inhibitors with a galloyl moiety, such as compound **5**, had more interactions with the binding pocket. This may explain the improved potency of these compounds. The configuration of the benzene ring B (Fig. 1A) also seems to have impact on the interactions and poses. For smaller inhibitors, it might be more important than for the larger ones to reach partially the acetylated substrate binding site to gain inhibition activity.

Contrary to the inhibitors, activators bound outside of the cleft and formed interactions to the β6/α6 loop which is a part of a stable antiparallel three-stranded β sheet motif forming the acetyl-lysine binding tunnel. The importance of this β6/α6 loop area for substrate binding for all sirtuins has been discussed previously^43,44^. Thus, the activators might improve the binding of acetylated substrates by inducing conformational changes in the β6/α6 loop when binding to the putative activator site. Known activators, oleic and linoleic acids bound between the inhibitor binding site and the N-terminal tail. These fatty acids as well as anthocyanidins induced changes in the β6/α6 loop conformation or orientation of residues in this loop. The observed dual role of some flavonoids might be explained by their ability to bind both inhibitor and activator sites. Based on the docking results, these dual modulators can also form interactions with the same amino acid residues as do the best activators and inhibitors. Taken together, the observed dual role of some flavonoids, such as compound **14**, might be explained by their ability to bind multiple sites and form similar interactions as did the most potent modulators. However, the effect of these compounds is concentration dependent and thus, the mechanism of modulation might be more complex than with the activators and inhibitors. Therefore, it can be difficult to evaluate the reason for dual role of some flavonoids with docking or other studies.

To predict the possibility of the key residues at activator site (Gly156, Asp185, Trp186, Glu187 and Asp188) to have an impact on SIRT6 function or structure, an *in silico* based mutation analysis was carried out. The predictions (Suppl. Tables S3 and S4) show that these residues might be important SIRT6 function or structure. Future experiments, should target these residues for increased activity.

Previously, it has been demonstrated that some flavonoids^14,45–50^, including quercetin and luteolin, were SIRT6 modulators. This study included a larger array of molecular diverse structures of polyphenols and identified other classes of flavonoids with robust SIRT6 activity. Additionally, this study demonstrated that different classes of flavonoids can either inhibit or activate the deacetylation activity of SIRT6. Moreover, the effect was found to be dependent on the flavonoid subclass: catechins showed inhibition, anthocyanidins activation and flavonones and flavonols both showed inhibition and activation of SIRT6. Molecular modeling studies, also revealed discrete putative binding sites for both inhibitors and activators.

## Methods

### Materials

Acetylated histone H3(K9) peptide (residues 1–21) (H3K9Ac) was from AnaSpec (USA). Fetal bovine serum (FBS), Novex 10–20% gradient gels, anti-GLUT1-IgG (PA5–16793), anti-SIRT6-IgG (PA517215) and anti-rabbit-IgG (mouse) Horseradish peroxidase (HRP)-conjugated secondary antibody (G21234) were from Life Technologies (UK). Anti- FoxO3α-IgG (SAB3500508), anti-*α*-Tubulin (T5168)-IgG1, anti-Twist1-IgG (SAB2106420), NAD^+^, formic acid and compounds were from Sigma Aldrich (USA). Anti-mouse-IgG (rabbit) HRP-conjugated secondary antibody (ab97046) was from Abcam (UK). Dulbecco modified Eagle medium (DMEM) and non-essential amino acids were from Lonza (Belgium). Rabbit anti-acetyl H3K9 antibody and purified chicken core histones (13–107) were from Millipore (USA). Enhanced chemiluminescence (ECL) prime western blotting detection reagents were from Amersham BioSciences (UK). Penicillin/Streptomycin was from EuroClone (Italy).

The human SIRT6 expression vector hSIRT6-pGEX-6P3 was kindly provided by Prof. Katrin Chua (Stanford, USA). Recombinant GST-tagged SIRT6 was produced by fermentation in *E*. *coli* BL21(DE3)-pRARE. The production was done at +16 °C with 0.1 mM IPTG for 20 h and the soluble overexpressed protein was purified on glutathione agarose (Sigma, Saint Louis, USA).

Radioimmunoprecipitation assay (RIPA) lysis buffer was prepared in 50 mM Tris-HCl buffer (pH = 8.0) consisting of 150 mM NaCl, 1% NP-40, 0.5% Na-deoxycholate, 5 mM EDTA, 0.1% SDS.

### SIRT6 deacetylation assay

The *in vitro* assay was carried out as previously described^14^. Briefly, 0.6 µl of compounds (100 µM) in DMSO and DMSO (control) were incubated for 30 min with GST-SIRT6 (3 µg/well), H3K9Ac (40 µM for activation/200 µM) and 500 µM NAD^+^ in Tris Buffer [25 mM, pH 8.0] at +37 °C. DMSO concentration was 1% in all samples. Control samples for compounds without NAD^+^ or SIRT6 were carried out. The deacetylation reaction was terminated by adding 6 µl of cold 10% formic acid and centrifuged for 15 min. The samples were analyzed by reversed-phase HPLC. The formation of deacetylated product (H3K9) and substrate (H3K9Ac) peaks was monitored and subsequently quantified by measuring area under the curve. The dose response was determined for compounds **4**, **5**, **13**, **15**, **16**, **17** (Fig. 1B) and **18** (0.5 dilutions from 1000 µM). Maximal activation for the most potent activators was determined by maximal effect indicating the maximal increase in activation for the tested compounds. Experiments were repeated in triplicate, and *IC*_50_/*EC*_50_ values were calculated using Graph Pad Prism Software version 6 (California, USA).

### HPLC

Chromatographic separation of H3K9/H3K9Ac was achieved on a Zorbax Eclipse XDB-C18 column (4.6 mm × 50 mm, 1.8 *µ* Agilent, Santa Clara, CA, USA) using a Shimadzu prominence system (Shimadzu, Japan) consisting of a CBM-20A, LC-20AB binary pumps, SIL-20AC-HT autosampler and DGU-20A3 degasser. Mobile phase consisted of water with 0.02% formic acid (eluent A) and acetonitrile with 0.02% formic acid (eluent B). The gradient of eluent B was set up as follows: 0–2.0 min 0%; 2.0–10 min 0–8%; 10–10.1 min 8–80%; 10.1–12 min 80%; 12–12.1 min 80–0%; 15 min 0%. Flow rate was 0.9 ml/min, run time 15 min and injection volume 20 *µ*l. HPLC system was coupled to a 5500 QTRAP equipped with Turbo V electrospray ionization source (TIS)® (Applied Biosystems, Foster City, CA, USA). Data were acquired and analyzed using Analyst version 1.5.1 (Applied Biosystems). Positive electrospray ionization data were acquired using multiple reactions monitoring (MRM). TIS instrumental source settings for temperature, curtain gas, ion source gas 1 (nebulizer), ion source gas 2 (turbo ion spray), entrance potential and ion spray voltage were 550 C, 20 psi, 60 psi, 50 psi, 10 V and 5500 V, respectively. TIS compound parameter settings for declustering potential, collision energy, and collision cell exit potential were 231 V, 45 V, and 12 V, respectively, for H3K9Ac and 36 V, 43 V and 12 V, respectively for H3K9. The standards were characterized using the following MRM ion transitions: H3K9Ac (766.339 → 760.690) and H3K9 (752.198 → 746.717).

### Western blot analysis

Western blot analysis was carried out as previously described, with slight modifications^20^. Briefly, 100 µM cyanidin or dephinidin and DMSO control were incubated for 30 min in the presence of 3 µg of purified recombinant GST-SIRT6, 1.25 µg purified chicken core histones, and 500 µM NAD^+^ in 25 mM Tris-HCl, pH 8.0 at +37 °C. The reaction was stopped with Laemmli (sample buffer) and separated by SDS-PAGE using 10–20% gradient gels and transferred onto polyvinylidene difluoride (PVDF) membranes. H3K9 acetylation was detected with rabbit anti-acetyl H3K9 antibody followed by anti-rabbit HRP-conjugated secondary antibody. Membranes were stripped and re-probed with rabbit anti-histone H3 antibody. Chemiluminescent signal detection and image acquisition were carried out using ECL prime western blotting detection reagents.

### Molecular modeling

In docking studies, Maestro 11.0.015 was used (Small-Molecule Drug Discovery Suite 2016-4, Schrödinger, LLC, New York, NY, 2016).

#### Protein structure preparation

SIRT6 protein structure was downloaded from RCSB Protein Data Bank (PDB ID: 3ZG6)^5^. The structure was prepared using Protein Preparation Wizard with default settings (assign bond orders, add hydrogens, create zero-order bonds to metals, create disulfide bonds). Hydrogen bonds were assigned using PROPKA (pH 7.4) and waters having less than 3 H-bonds to non-waters were removed (default setting). Protein was minimized using OPLS3 force field (heavy atom converging RMSD 0.30 Å). Myristoyl peptide was removed.

#### Ligand preparation

3D-structures for linoleic acid, oleic acid and compounds **1**–**27** were generated using Maestro and prepared with LigPrep using OPLS3 forcefield. Compounds were ionized at target pH 7.4, desalted and tautomers were generated using Epik. For stereoisomer generation, chiralities were determined from 3D structure.

#### Binding site detection

Inhibitor binding site was determined based on the binding site of SIRT1 inhibitor Ex-527 in crystal structure of SIRT1 (PDB ID 4I5I)^51^. SIRT6 and SIRT1 structures were overlaid, and binding site corresponding to Ex-527 in SIRT1 was determined for SIRT6. In this study, Maestro SiteMap was used to determine the activator binding site. At least 10 site points per reported site was set to be required, and shallow binding sites were also detected. Other settings were kept as default (identify top-ranked potential receptor binding sites, use more restrictive definition of hydrophobicity, use standard grid and crop site maps at 4 Å from nearest site point).

#### Docking

Ligands were docked with Maestro Induced Fit (Version 3.1, Glide Grid generation version 5.1) which docks ligands to the defined receptor with Glide, then Prime Refinement processes predefined amount of best scoring poses and relaxes the receptor structure. The ligands are redocked with Glide and the best scoring poses are shown. Herein, the grid center of inhibitor binding site was in the middle of Ile59, Phe62, Val68, Asn112 and Ile183, close to the binding site of nicotinamide. The grid center for the activator site (for control fatty acids and SIRT6 activators) was in the center of Ser8, Gly156, Lys158 and Glu187. Other settings were kept as default (grid enclosing box size 26 Å on a side, ring conformation sampling with energy window of 2.5 kcal/mol, penalize nonpolar conformation of amide bonds, receptor and Van der Waals scale of 0.5 Å, refine residues within 5.0 Å of ligand poses, redock within 30.0 kcal/mol of the best structure and use Standard Precision docking). The final poses selected for interaction comparison were among the best three scoring poses.

### *In silico* mutation analysis

The functional impact of the key SIRT6 residues at the putative activator site was assessed using SIFT (http://sift.bii.a-star.edu.sg/)^52^, PROVEAN (http://provean.jcvi.org/index.php)^53^ and PolyPhen-2 (http://genetics.bwh.harvard.edu/pph2/)^54^. PROVEAN and SIFT employ sequence similarity based methods to identify homologous sequences and use the sequence conservation to calculate impact score(s). PolyPhen-2 also uses sequence-based information but, in addition uses 3D structure based predictive features. SIRT6 genomic and residue information were obtained from Ensembl transcript (ENST00000337491.6), and UniProt (ID: Q8N6T7-1) respectively. *In silico* alanine type scanning was used to assess the functional importance of the key residues. The amino acid substitutions were either neutral (Ala), or other residues that possess drastically different chemical properties. The results (Suppl. Table S3) predict, whether residues are likely to be important either for the function or structure of SIRT6.

### Cell culture and treatments

Caco-2 cells (passage 30–40) were cultured in DMEM with 10% FBS, 1% nonessential amino acids, 2 mM L-glutamine, 100 units/ml of penicillin, and 100 *µ*g/ml of streptomycin at +37 °C. Cells were cultured 14 days before the treatments. For the immunoblotting, cells were seeded on 24-well plates (1 × 10^5^ cells/well). After 24 hours, cells were treated with 0.5% DMSO (control) or various concentrations (12.5–200 *µ*M) of compound **17** for 24 h.

### Preparation of total cell fraction for immunoblotting

After treatment, medium was discarded and cells were washed twice with ice-cold PBS. RIPA buffer was added to the cells and incubated for 30 min. Cell suspensions were collected and centrifuged (13,000 rpm, 20 min +4 °C). Supernatant containing the proteins were aliquoted and stored at −80 °C. Sample protein concentrations were measured with Bradford Assay (Bio-Rad DC^TM^ Protein Assay).

### Immunoblotting

Immunoblotting was performed according to standard protocols from four independent experiments. Briefly, protein samples (18 µg/sample) were separated by SDS-PAGE using 10–20% gradient gels and transferred onto PVDF membranes. Membranes were blocked in 3% non-fat dry milk and further incubated with primary rabbit anti- FoxO3α (1:2000), anti-GLUT1 (1:1000), anti-H3, anti-SIRT6 (1:2000), anti-Twist (1:2000) anti-α-tubulin (1:8000) antibodies overnight at +4 °C. HRP-conjugated secondary antibodies (goat anti-rabbit, goat anti-mouse) were incubated for 1 hour at room temperature and proteins were detected using ELC prime western blotting system. Densitometric analysis of protein bands was carried out using ImageJ 1.32 software and the data were normalized by α-tubulin (loading control).

## Electronic supplementary material

Supplementary Information
