# Supplementary material for: Natural polyphenols as sirtuin 6 modulators
Source: Sci Rep. 2018 Mar 7;8:4163. doi: 10.1038/s41598-018-22388-5 (PMC5841289; doi:10.1038/s41598-018-22388-5)
Supplement: Supplementary file 1 — Supplementary Information [file 41598_2018_22388_MOESM1_ESM.pdf]

Supplementary Information

Natural polyphenols as sirtuin 6 modulators

Minna Rahnasto-Rilla,[1,2,+] Jonna Tyni,[2,+] Marjo Huovinen[2], Elina Jarho[2], Tomasz Kulikowicz[1], Sarangan Ravichandran[3], Vilhelm A. Bohr[1], Luigi Ferrucci[1], Maija Lahtela-Kakkonen,*[,2] and Ruin Moaddel*[,1]

[1]Biomedical Research Center, National Institute on Aging, National Institutes of Health, Baltimore, Maryland 21224,
USA
[2]School of Pharmacy, University of Eastern Finland, P.O.Box 1627, 70210, Kuopio, Finland
[3]Advanced Biomedical Computing Center, Frederick National Laboratory for Cancer Research, Leidos Biomedical Research Inc., Fredrick, Maryland 21701, USA
[*]maija.lahtela-kakkonen@uef.fi; Moaddelru@grc.nia.nih.gov
[+]these authors contributed equally to this work

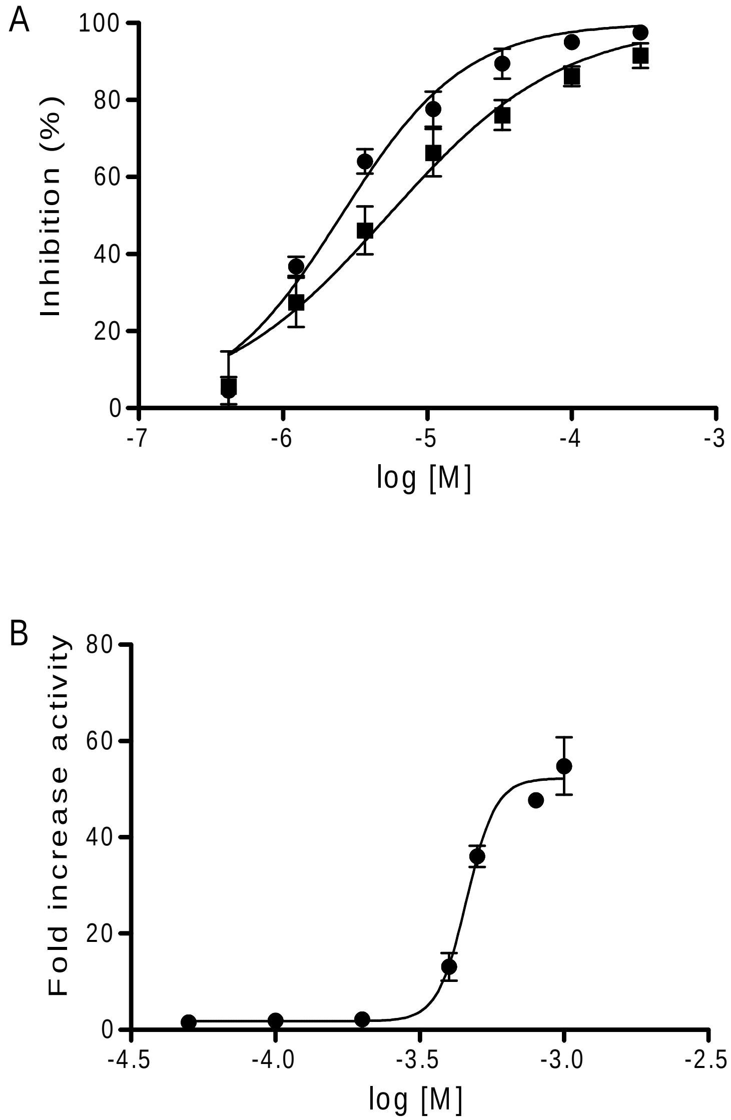

Figure S1. The IC_{50} curves for compounds **4** (●) and **5** (■) (A), and EC_{50} curve for compound **17** (B) with the standard deviation of the different measurements, n=3.

Table S1. SIRT6 activity data of flavonoids. The data is presented as means ± SD, n=3.

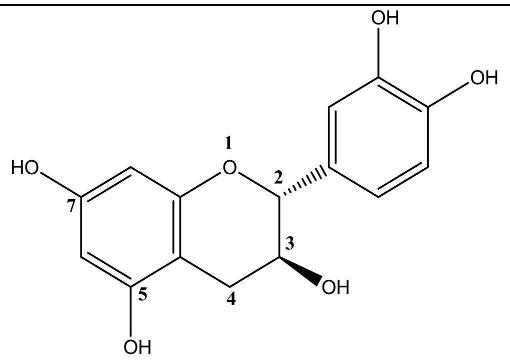

		Inhibition % 100 µM	Inhibition % 10 µM	Activation 100 µM
**1**	(+)-Catechin	26 ± 1.3	17 ± 1.6	0.8 ± 0.02

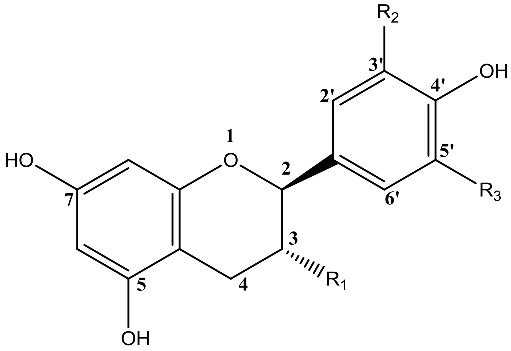

		R1	R2	R3	Inhibition % 100 µM	Inhibition % 10 µM	Activation 100 µM
**2**	(−)-Catechin	OH	OH	H	54 ± 0.5	25 ± 0.8	0.4 ± 0.04
**3**	(−)-Gallocatechin	OH	OH	OH	23 ± 1.0	17 ± 0.8	0.2 ± 0.01
**4**	(−)-Catechin gallate	gal[a]	OH	H	88 ± 0.2	62 ± 0.7	0.1 ± 0.03
**5**	(−)-Gallocatechin gallate	gal[a]	OH	OH	84±0.6	79±0.5	0.01 ± 0.01

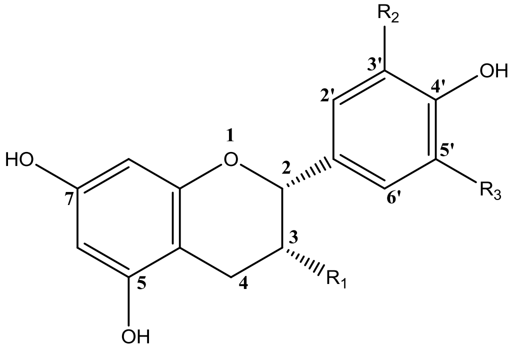

		R1	R2	R3	Inhibition % 100 µM	Inhibition % 10 µM	Activation 100 µM
**6**	(−)-Epicatechin	OH	OH	H	10 ± 0.1	nd	nd
**7**	(−)-Epigallocatechin	OH	OH	OH	4.1 ± 0.1	nd	nd
**8**	(−)-Epicatechin gallate	gal[a]	OH	H	60 ± 3.6	15 ± 0.9	0.1 ± 0.01
**9**	(−)-Epigallocatechin gallate	gal[a]	OH	OH	42 ± 1.9	10 ± 1.4	0.8 ± 0.04

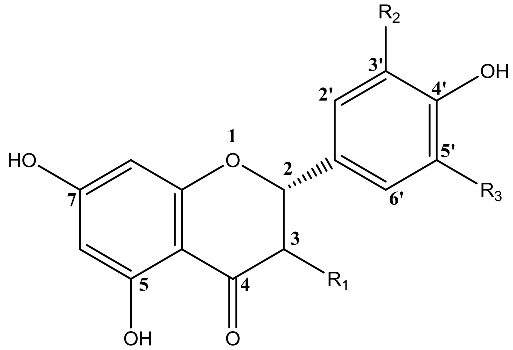

		R_1	R_2	R_3			
**10**	Naringenin	H	H	H	23 ± 1.5	8.3 ± 1.1	1.1 ± 0.05
**11**	Eriodictoyl	H	OH	H	27 ± 1.5	19 ± 2.2	1.1 0.2

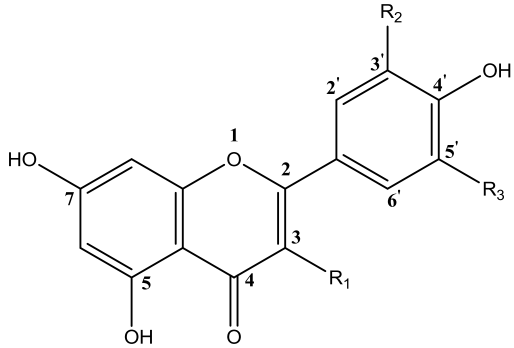

		R_1	R_2	R_3			
**12**	Apigenin	H	H	H	1.0 ± 0.6	nd	0.9 ± 0.01
**13**	Luteolin	H	OH	H	29 ± 0.3	24 ± 1.8	1.2 ± 0.04
**14**	Kaempferol	OH	H	H	24 ± 1.7	40 ± 0.8	2.2 ± 0.1
**15**	Quercetin	OH	OH	H	40 ± 3.0	27 ± 0.4	1.5 ± 0.1
**16**	Myricetin	OH	OH	OH	0	nd	2.3 ± 0.2

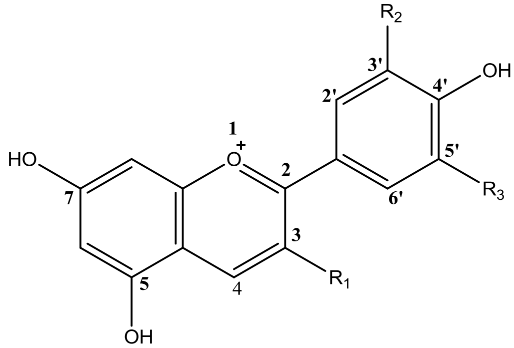

		R_1	R_2	R_3			
**17**	Cyanidin	OH	OH	H	0	nd	2.6 ± 0.1
**18**	Delphinidin	OH	OH	OH	0	nd	2.2 ± 0.1

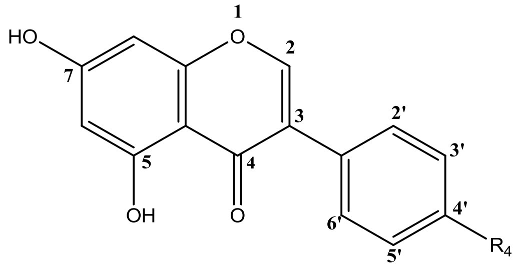

		R_4			
**19**	Genistein	OH	9.6 ± 1.4	1.8 ± 0.3	0.7 ± 0.2
**20**	Biochanin A	OCH_3	24 ± 0.9	13 ± 0.5	1.3 ± 0.02

Table S2. SIRT6 activity data of phenolic acids. The data is presented as means ± SD, n=3.

	**Compound**	**Inhibition %** 100 µM	**Activation** 100 µM
**21**	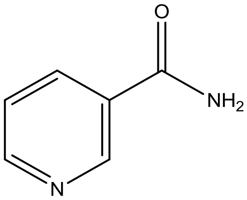 Nicotinamide	58 ± 1.3[a]	nd
**22**	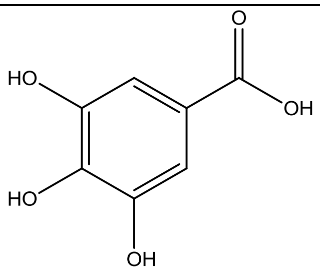 Gallic Acid	14 ± 1.2	1.4 ± 0.04
**23**	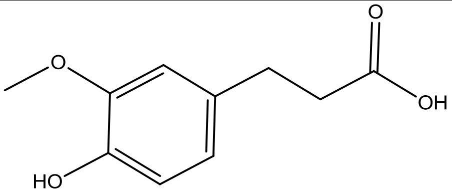 3-(4-Hydroxy-3-methoxyphenyl)propionic acid	-12 ± 1.1	1.8 ± 0.05

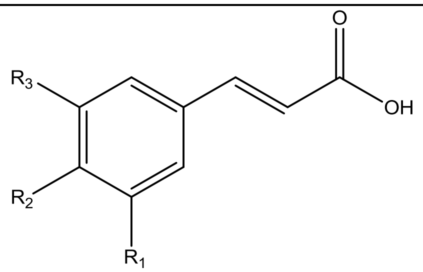

		R_1	R_2	R_3		
**24**	p-Coumaric acid	H	OH	H	8.5 ± 1.2	0.6 ± 0.1
**25**	Caffeic Acid	OH	OH	H	-5.9 ± 0.2	1.0 ± 0.01
**26**	trans-Ferulic acid	OCH_3	OH	H	-8.6 ± 0.3	0.9 ± 0.6
**27**	Sinapic Acid	OCH_3	OH	OCH_3	8.0 ± 0.9	0.6 ± 0.2

[a] 1000 µM concentration

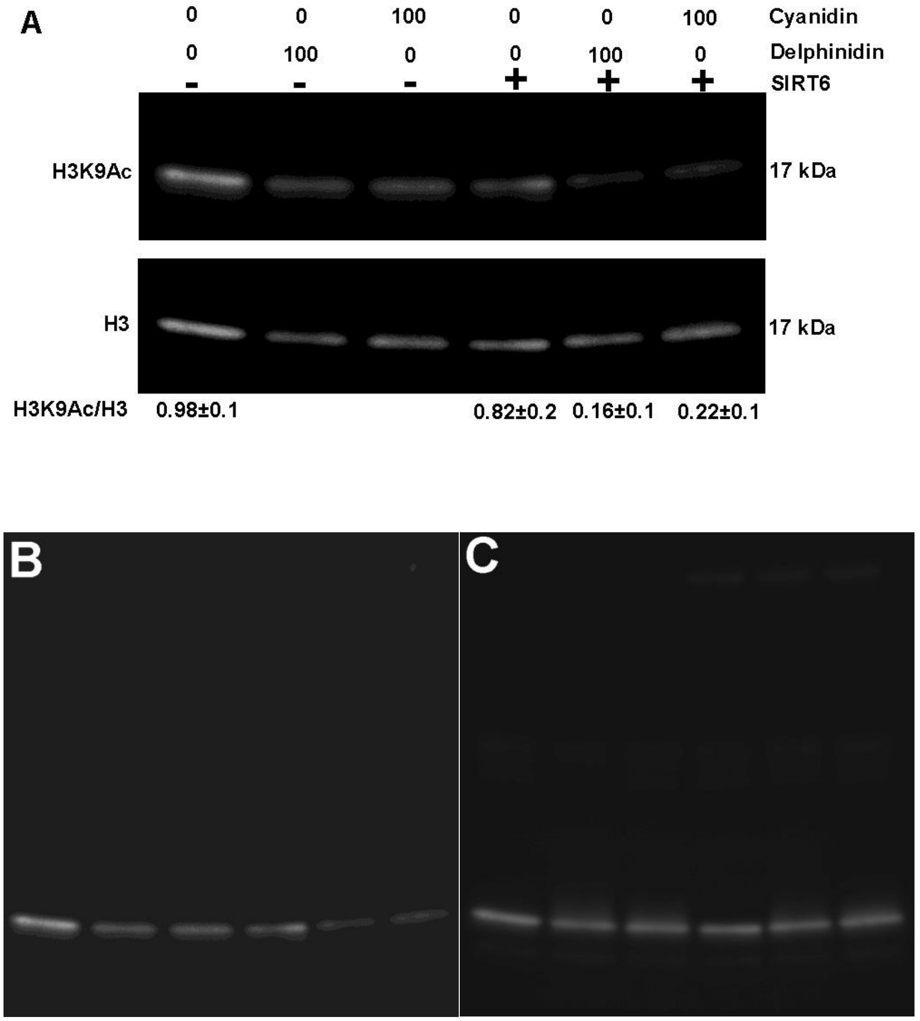

Figure S2. Immunoblotting analysis of H3K9 at the concentration of 100 µM delphinidin and cyanidin, 3 µg/well of a purified recombinant GST-SIRT6 protein, 1.25 µg purified whole chicken core histones with 500 µM NAD^+ in 25 mM Tris-HCl, pH 8.0. Analysis were repeated three times and one representative cropped blot is shown (A**)**. Uncropped blots probed with H3K9Ac (B), H3 (C).

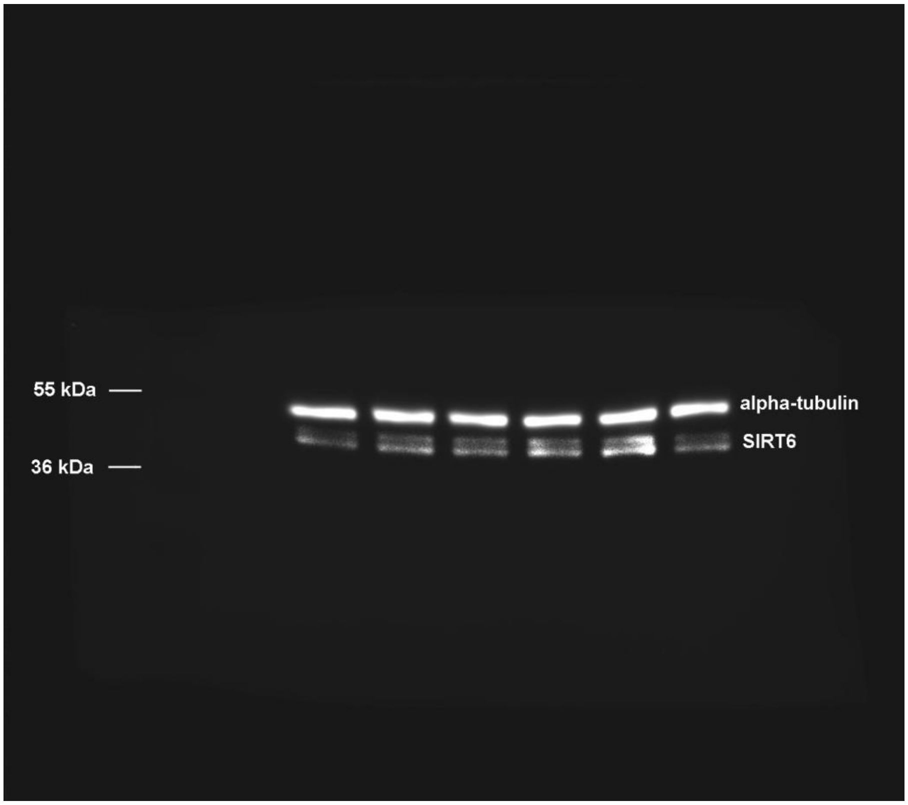

Figure S3. Uncropped blots probed with SIRT6 and α-tubulin in Figure 3B.

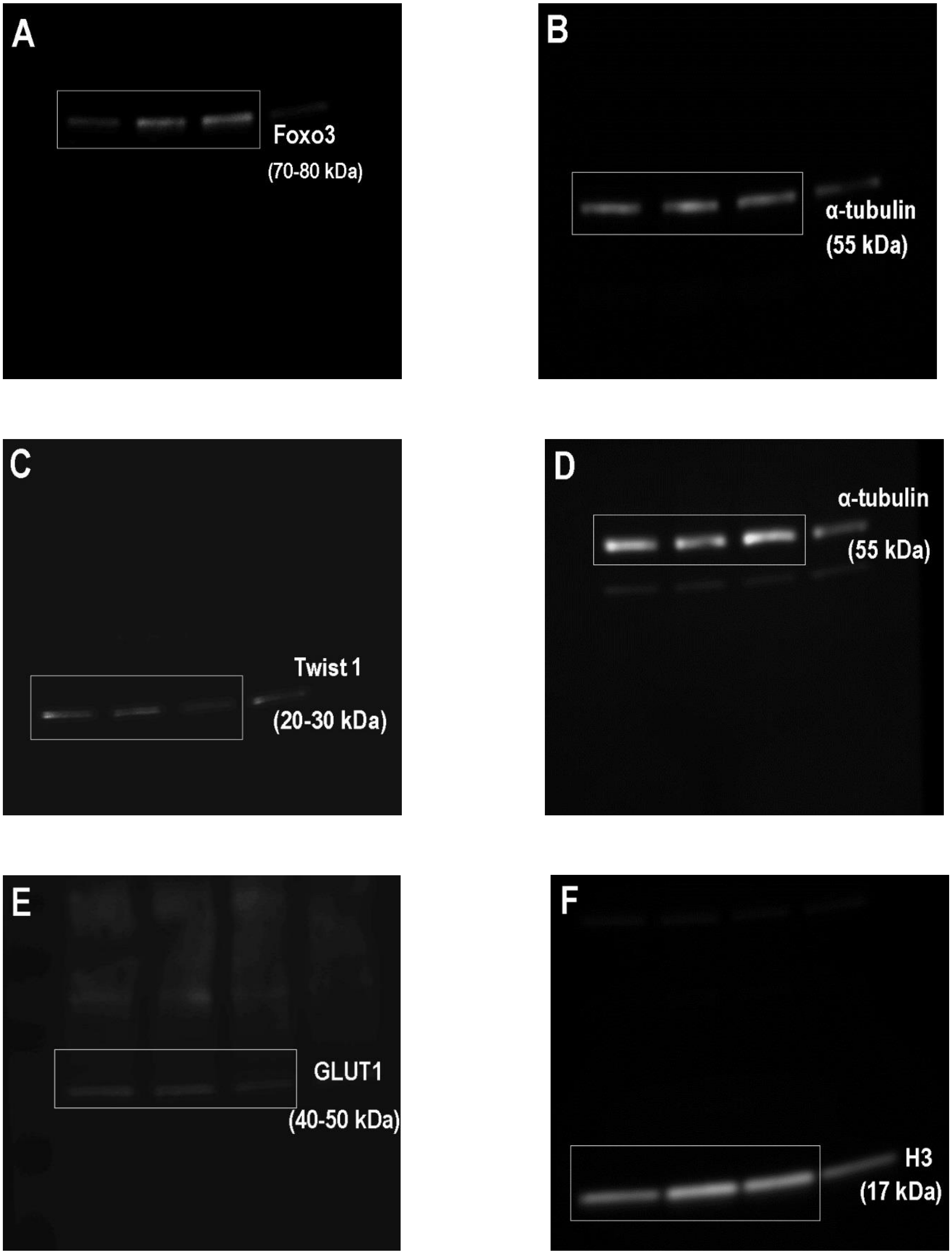

Figure S4. Full blot images for Figure 4 with approximate regions used for figures marked with rectangles.

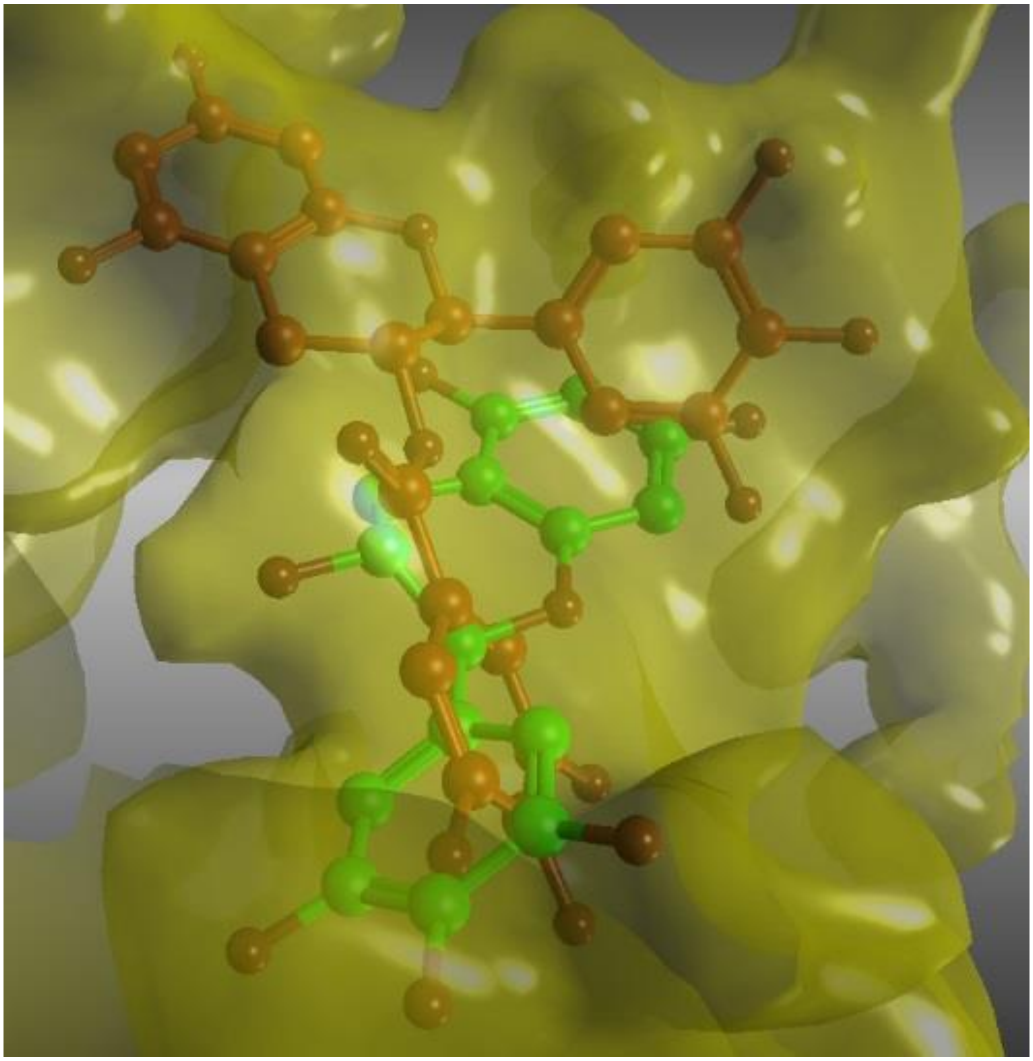

Figure S5. The complementary filling of compound **3** (green) and **5** (brown) in the inhibitor binding pocket of SIRT6. The yellow surface represents the inhibitor binding pocket.

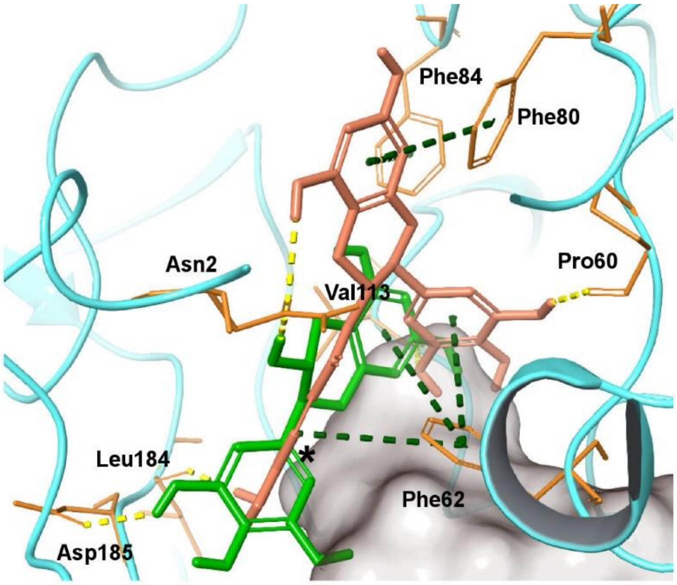

Figure S6. Aligned docking poses of compounds **3** (green) and **5** (brown) at SIRT6 inhibitor site. Yellow dashes indicate hydrogen bonding and green dashes indicate π-π stacking to different amino acids (orange). Grey area represents NAD^+ binding pocket. Gallate-moiety of compound **5** is marked with *. For 2D interaction diagrams of compounds **3** and **5**, see Suppl. Fig. S11.

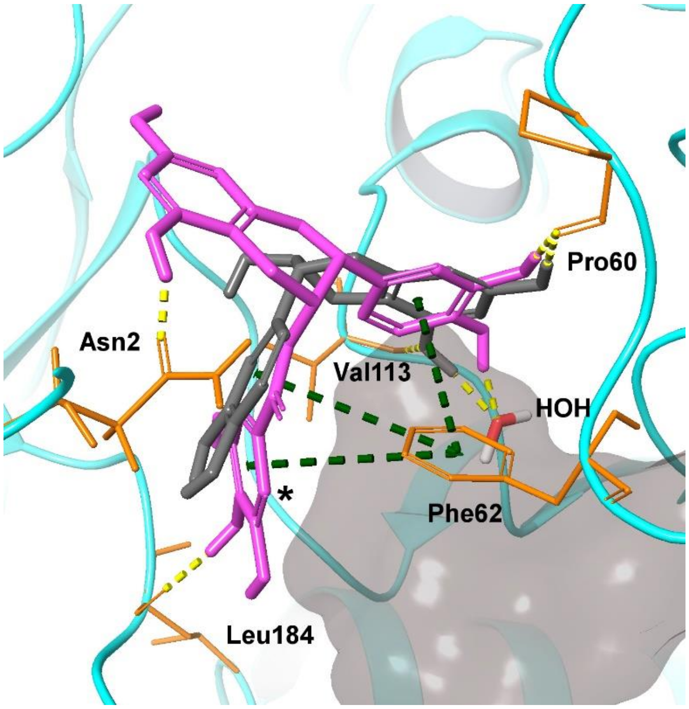

Figure S7. Aligned docking poses of compounds **6** (dark grey) and **8** (magenta) at SIRT6 inhibitor site. Yellow dashes indicate hydrogen bonding and green dashes indicate π-π stacking to different amino acids (orange). Grey area represents NAD^+ binding pocket. Gallate-moiety of compound **6** is marked with *. For 2D interaction diagrams of compounds **6** and **8**, see Suppl. Fig. S12.

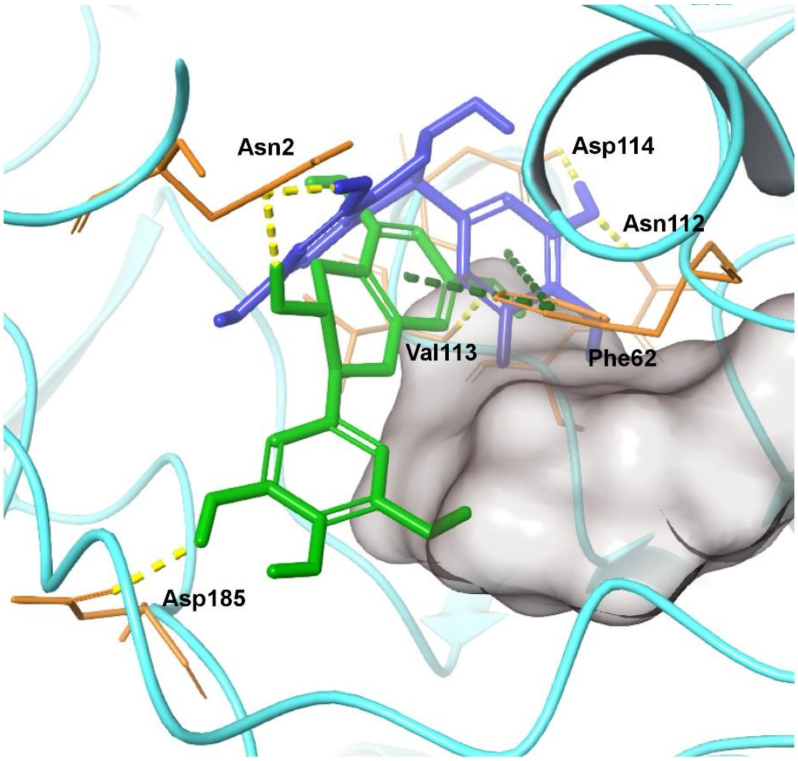

Figure S8. Aligned docking poses of compounds **3** (green) and **7** (blue) at SIRT6 inhibitor binding site. Yellow dashes indicate hydrogen bonding and green dashes indicate π-π stacking to different amino acids (orange). Grey area represents NAD^+ binding pocket. For 2D interaction diagram of compounds **3** and **7**, see Suppl. Fig. S11 and S12, respectively.

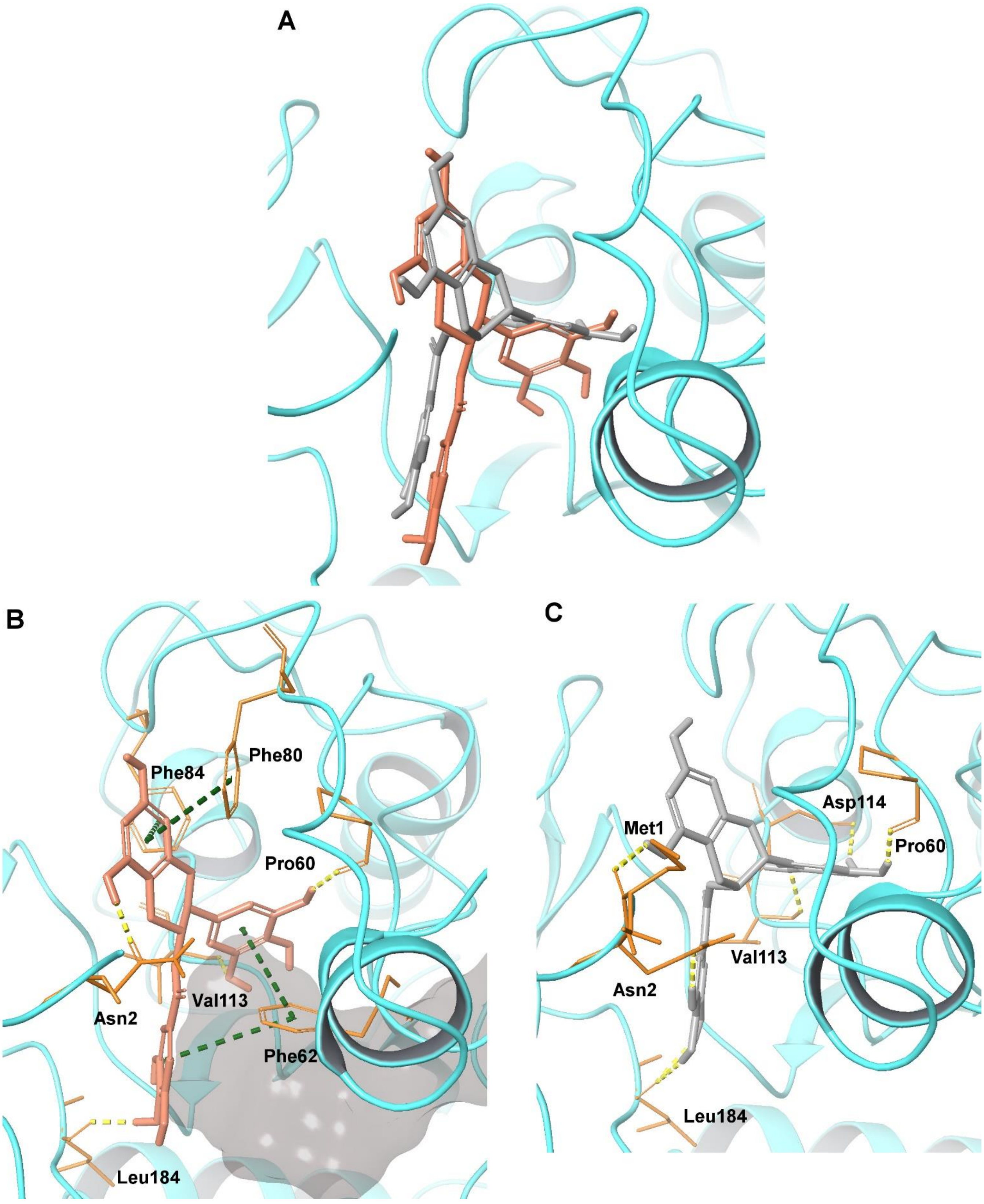

Figure S9. Aligned docking poses of compounds **5** and **9** at SIRT6 inhibitor binding site (A). Docking poses and interactions of compounds **5** (brown) (B) and **9** (grey) (C). Yellow dashes indicate hydrogen bonding and green dashes indicate π-π stacking to different amino acids (orange). Grey area represents NAD^+ binding pocket. For 2D interaction diagrams of compounds **5** and **9**, see Suppl. Fig. S11 and S12, respectively.

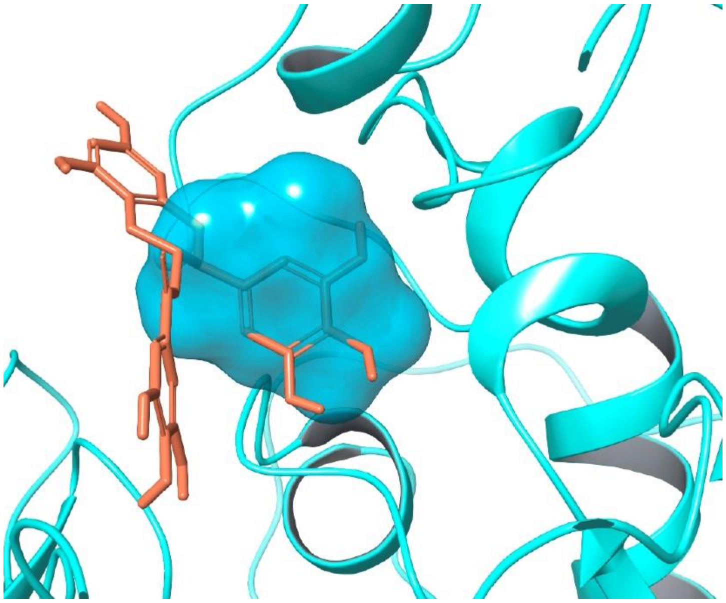

Figure S10. The location of the binding site (blue area) of phenolic acids at the inhibitor binding pocket compared to the binding area of inhibitor, compound **5** (brown).

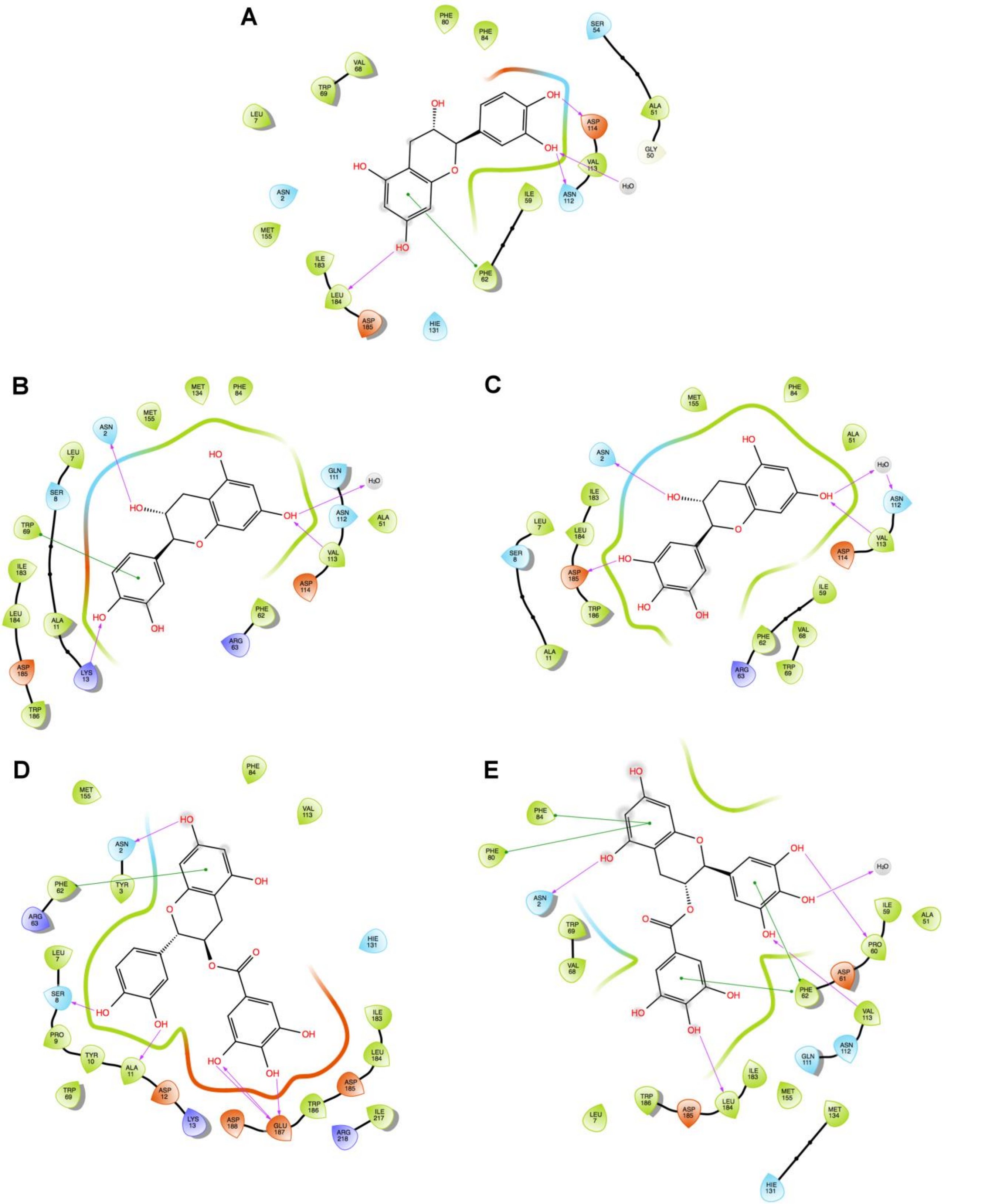

Figure S11. Docking poses of catechins at the inhibitor binding site: compounds **1** (A), **2** (B), **3** (C), **4** (D) and **5** (E). Purple arrows indicate hydrogen bonding, green lines indicate π-π stacking.

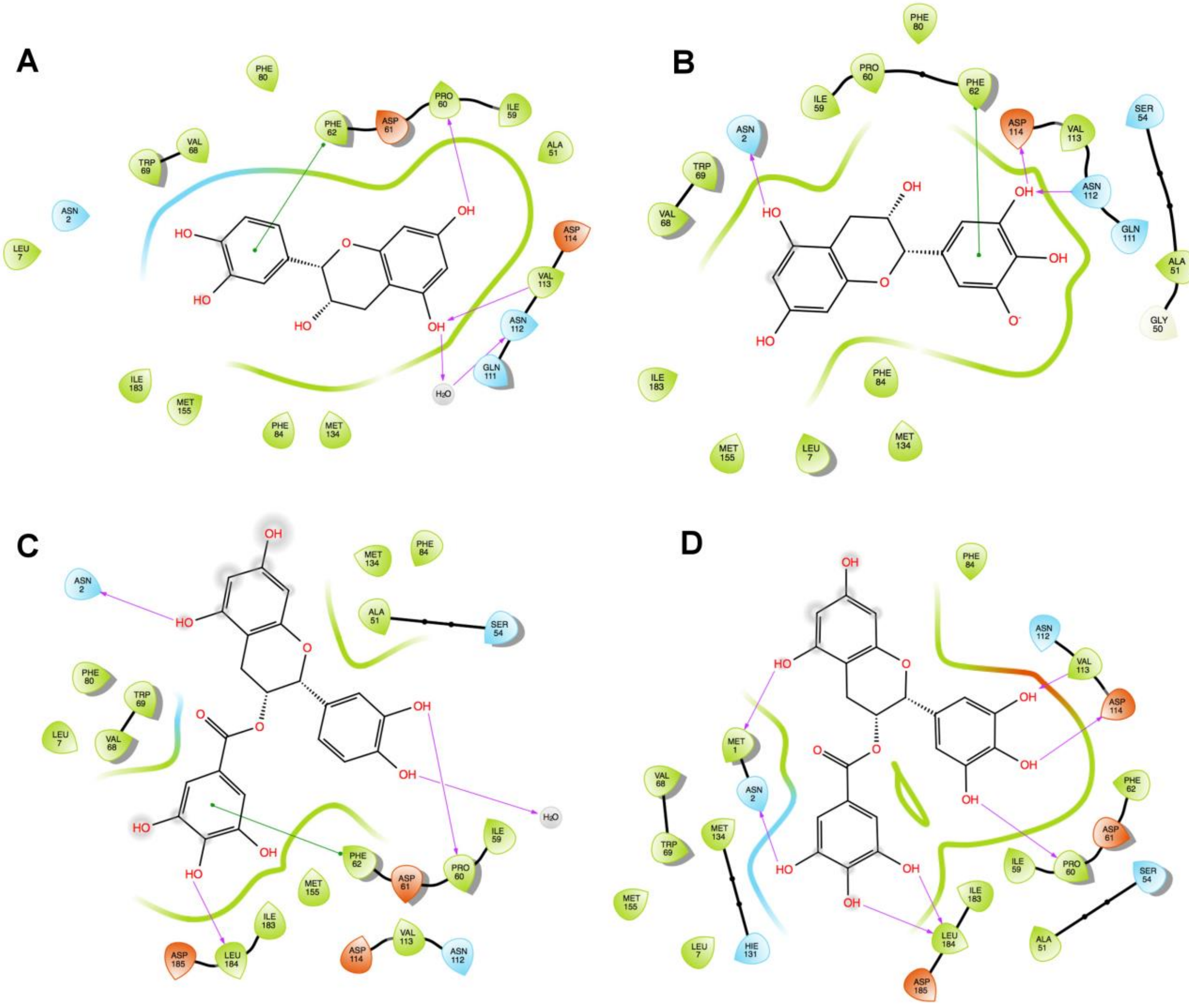

Figure S12. Docking poses of epicatechins at the inhibitor binding site: compounds **6** (A), **7** (B), **8** (C) and **9** (D).

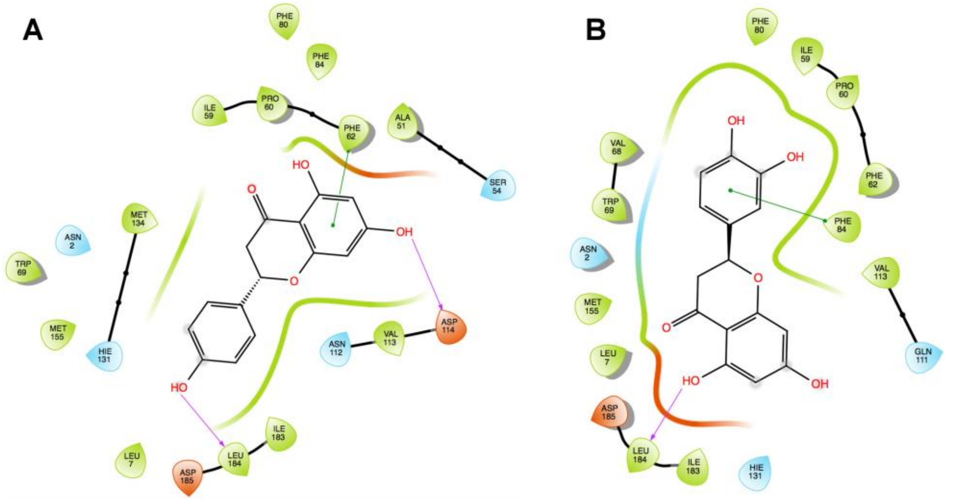

Figure S13. Docking poses of flavanones at the inhibitor binding site: compounds **10** (A) and **11** (B). Purple arrows indicate hydrogen bonding, green lines indicate π-π stacking.

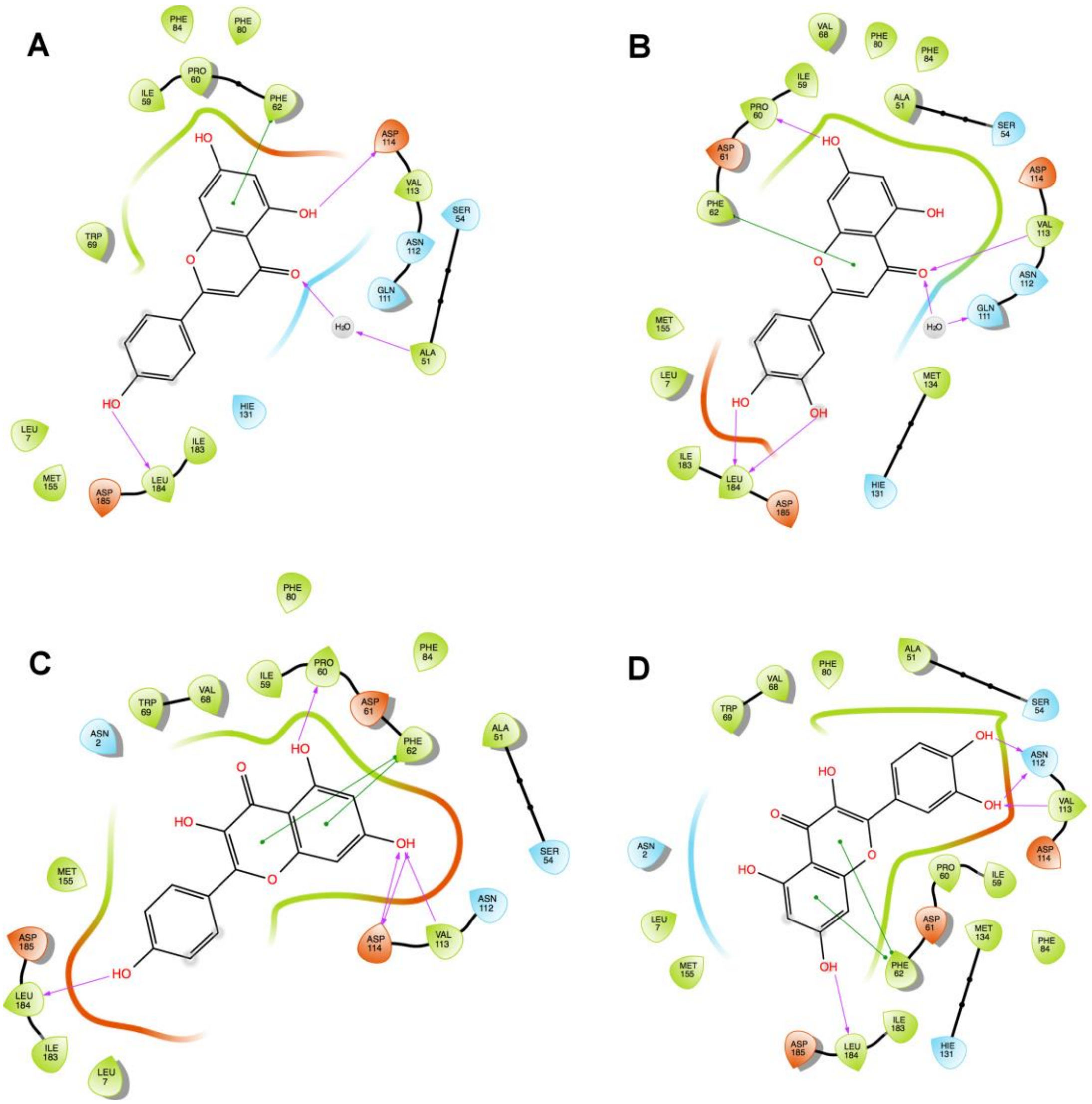

Figure S14. Docking poses of flavones at the inhibitor binding site: compounds **12** (A), **13** (B), **14** (C) and **15** (D). Purple arrows indicate hydrogen bonding, green lines indicate π-π stacking.

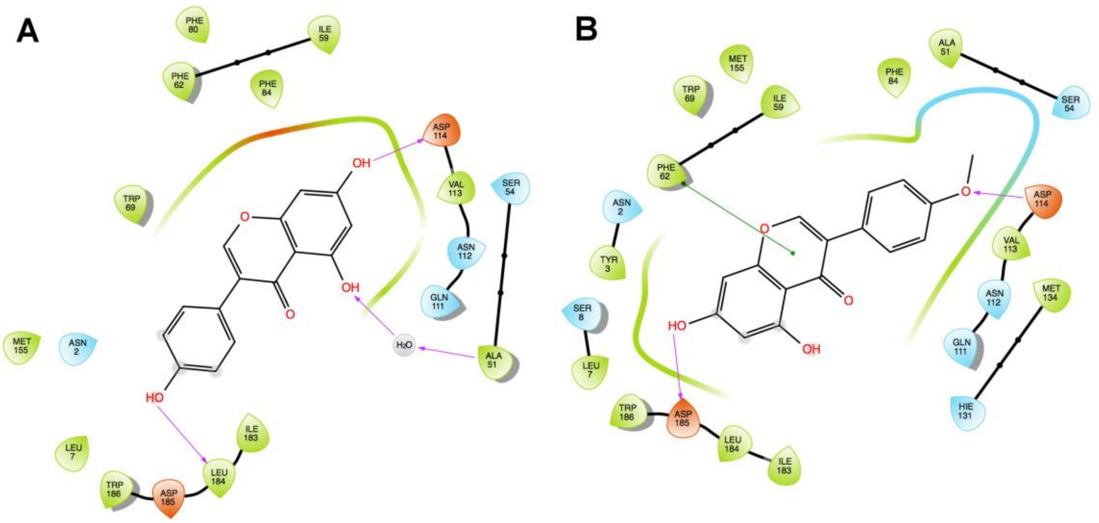

Figure S15. Docking poses of isoflavones at the inhibitor binding site: compounds **19** (A) and **20** (B). Purple arrows indicate hydrogen bonding, green lines indicate π-π stacking.

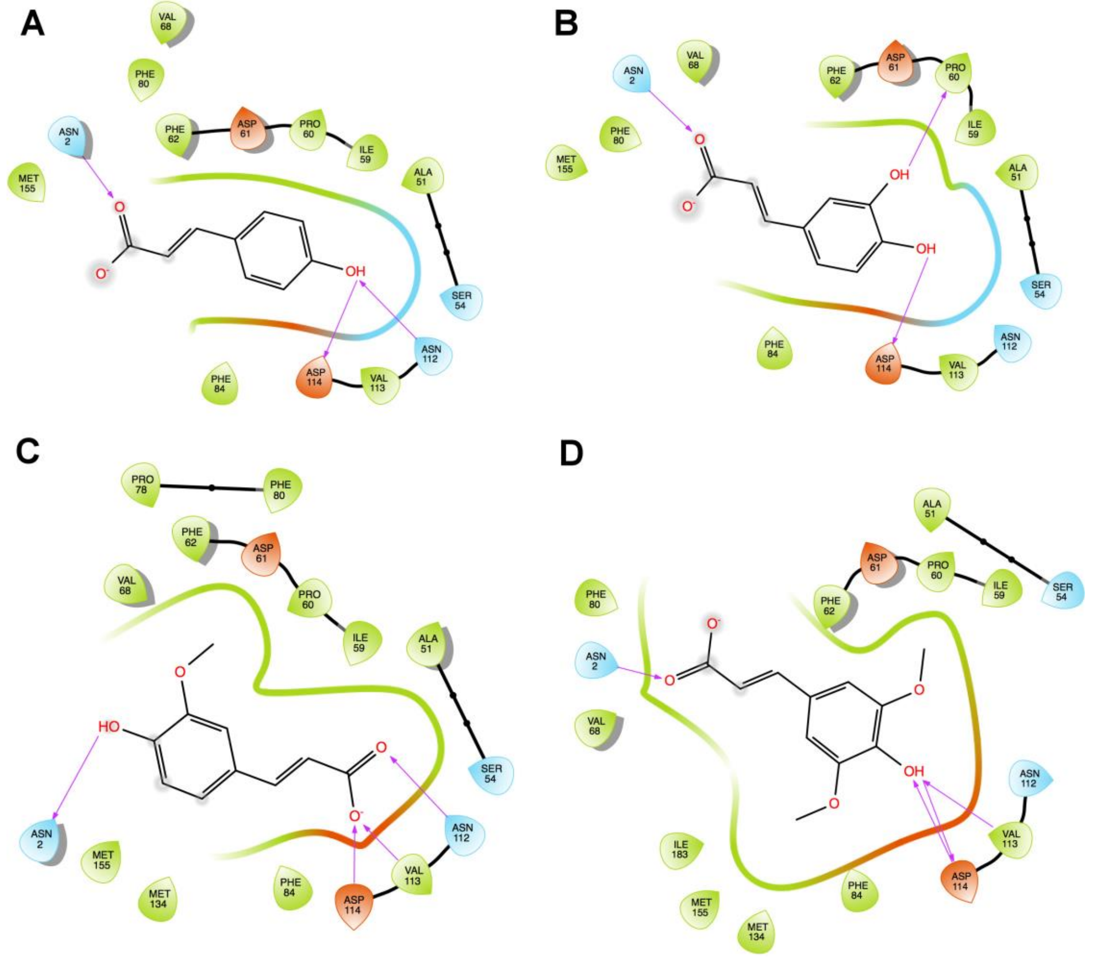

Figure S16. Docking poses of inactive phenolic acids at the inhibitor binding site: compounds **24** (A), **25** (B), **26** (C) and **27** (D). Compounds **22** and **23** formed also similar interactions at the inhibitor binding site (data not shown). Purple arrows indicate hydrogen bonding, green lines indicate π-π stacking.

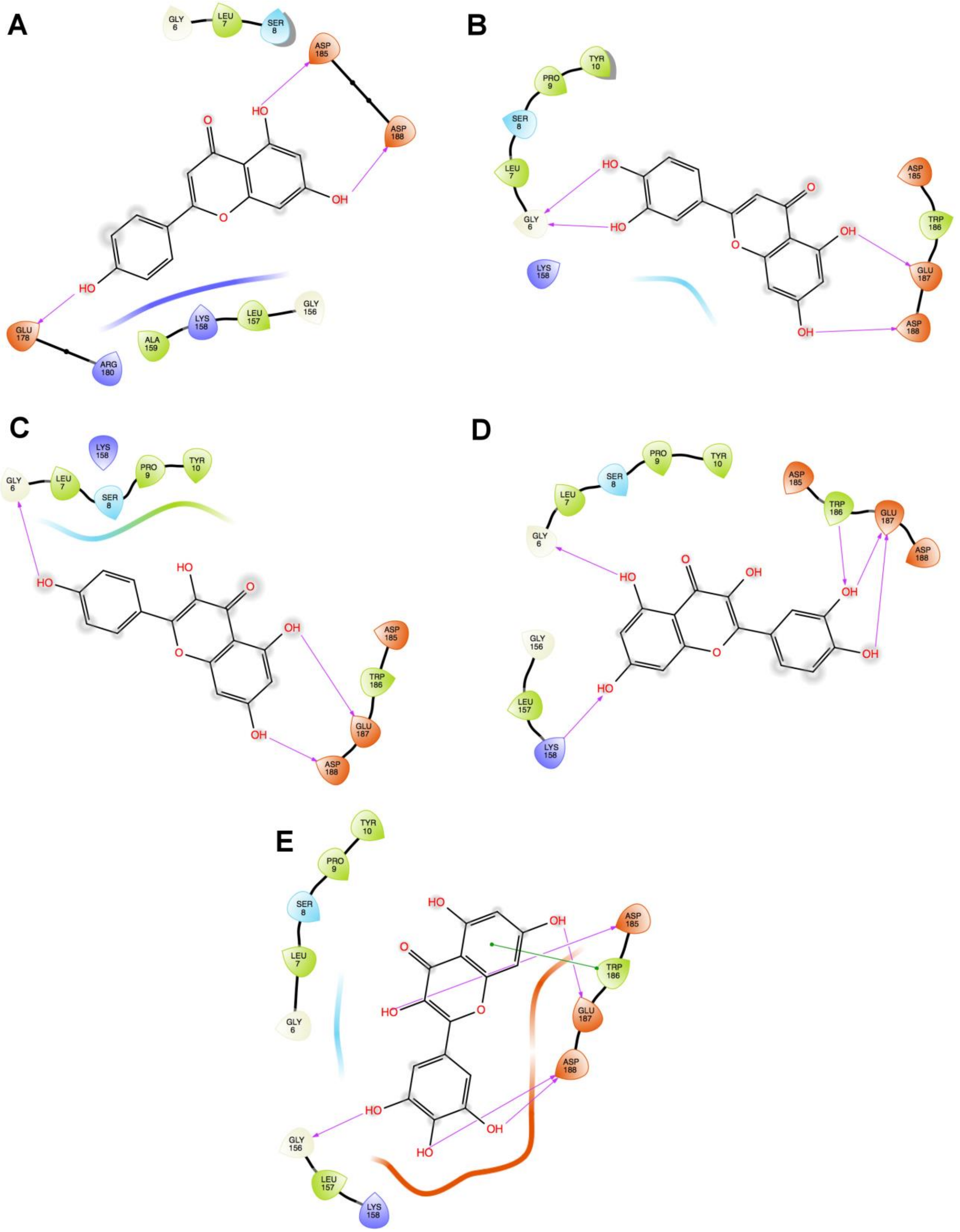

Figure S17. Docking poses of flavones at the activator binding site: compounds **12** (A), **13** (B), **14** (C), **15** (D) and **16** (E). Purple arrows indicate hydrogen bonding, green lines indicate π-π stacking.

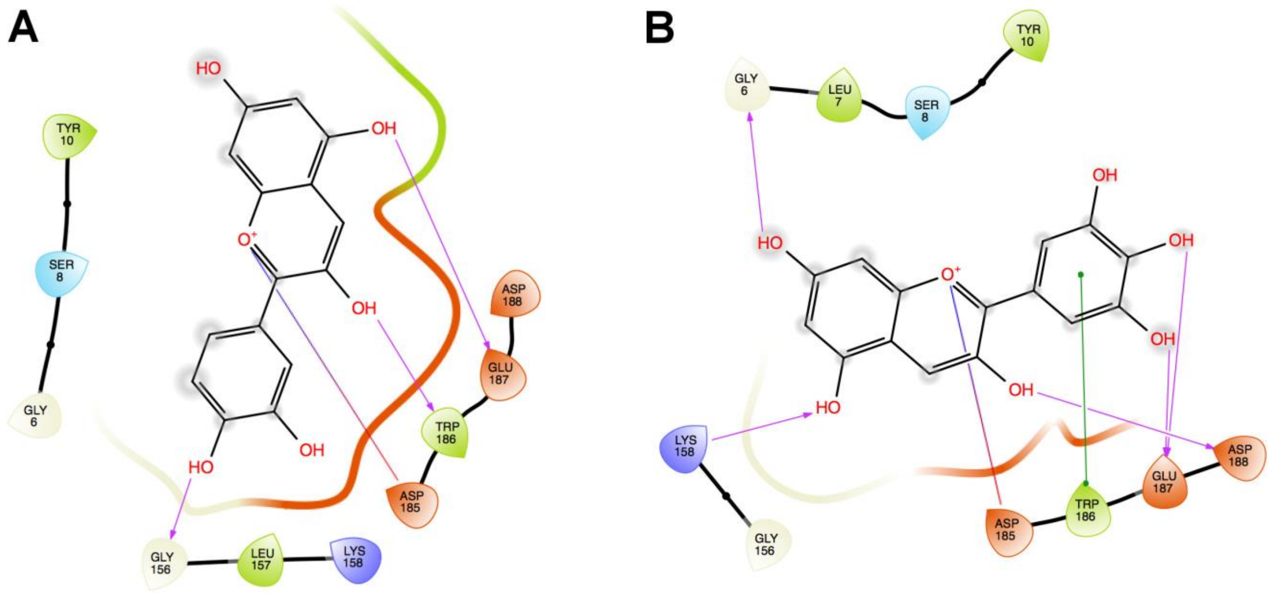

Figure S18. Docking poses of anthocyanins at the activator binding site: compounds **17** (A) and **18** (B). Purple arrows indicate hydrogen bonding, green lines indicate π-π stacking, blue-red lines indicate salt bridge.

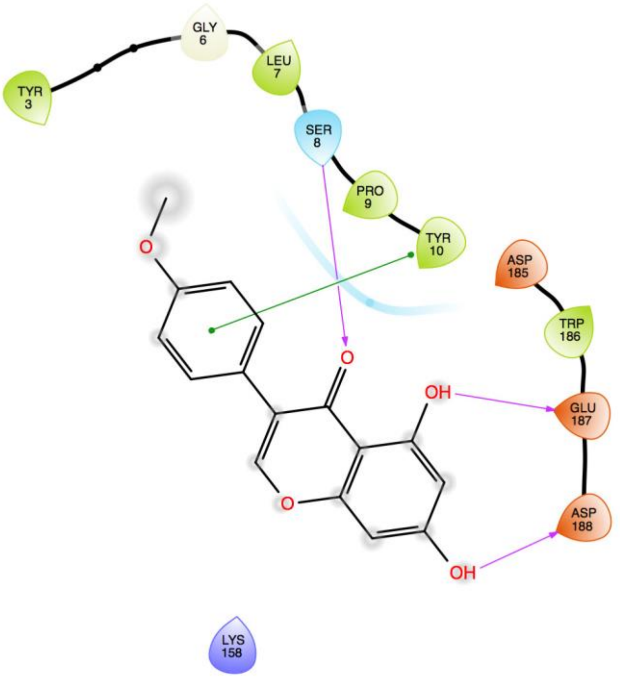

Figure S19. Docking pose of isoflavone, compound **20**, at the activator binding site. Purple arrows indicate hydrogen bonding, green lines indicate π-π stacking.

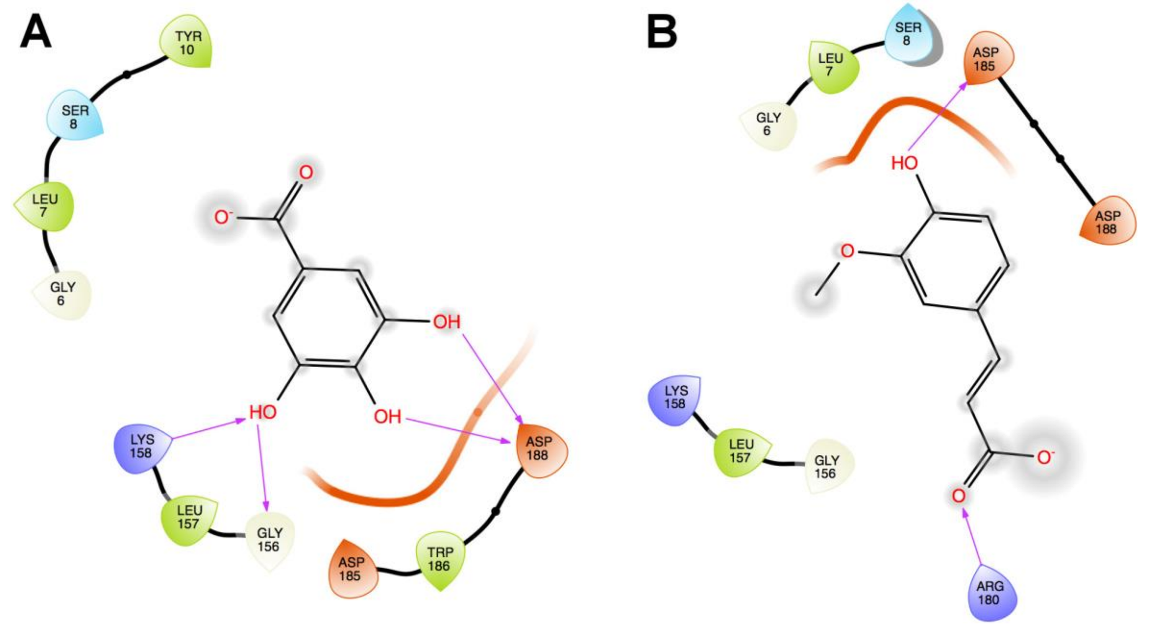

Figure S20. Docking poses of phenolic acids at the activator binding site: compounds **22** (A) and **26** (B). Other phenolic acids did not bind to the activator binding site in these docking studies. Purple arrows indicate hydrogen bonding, green lines indicate π-π stacking.

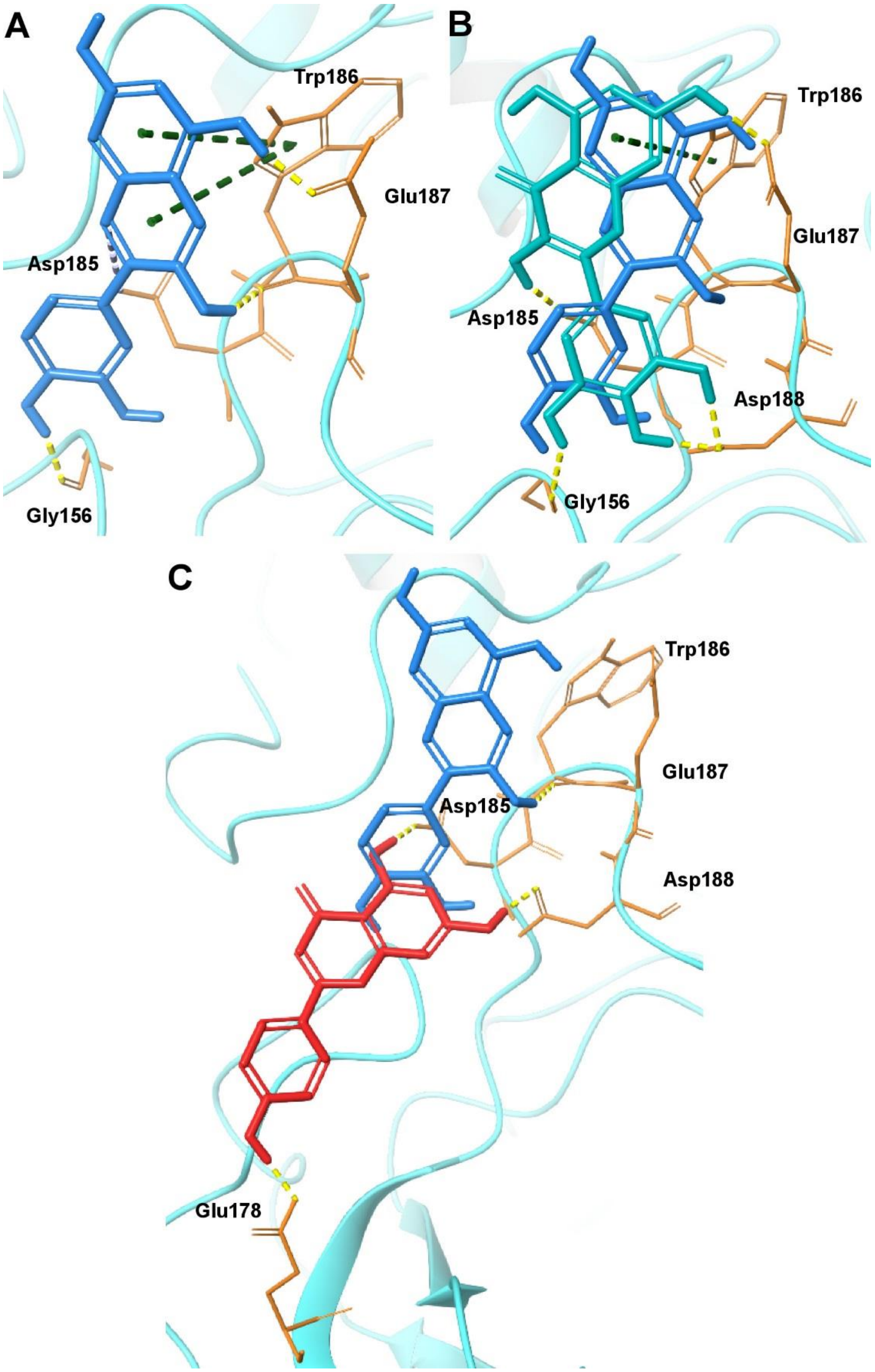

Figure S21. The pose and interactions of the most potent activator compound **17** at the SIRT6 putative activator binding site (A). The pose and interactions of potent activator compound **16** (turquoise) (B) and inactive compound **12** (red) (C) compared to the pose of compound **17** (blue). Yellow dashes indicate hydrogen bonding, green dashes indicate π-π stacking and light purple dash indicates salt bridge.

Table S3: SIFT Protein sequence based predictions. Threshold for intolerance was set to 0.05. Note that the capital letter amino acids shown in Predict Tolerated column are part of the sequence alignment, and the lower-case ones are from prediction. Sequence represented (Seq Rep) values report the fraction that contain one of the basic amino acids. Seq Rep was found to be 1.0 for all the variations.

Position	Predict Tolerated
**Gly156**	GLY
**Asp185**	ASP
**Trp186**	TRP
**Glu187**	ASP, GLU
**Asp188**	ALA, glu, LYS, ser, GLY, asn, HIS, ASP

Table S4: Variant impact assessments using sequence and structure based methods. Note that #SEQ denotes the number of sequences and #CLUSTER indicate the number of clusters used in the prediction. HumVar and HumDiv are two classifier models used by PolyPhen-2. HumDiv is preferred for evaluating rare variants and HumVar is more suitable for variants that could result in Mendellian diseases. MEDIAN INFO indicates the median information can be used to measure the diversity of the sequences. Sequence Databases used by PROVEAN, SIFT and PolyPhen-2 are NCBI nr Sep 2012, UniRef90 2011_08 and UniRef100 2011_12/PDB-DSSP Snapshot 03-01-2012 respectively.

List of other abbreviations used in the table: PRD: Probably Damaging, POD: Possibly Damaging, APF: Affect Protein Function, BEN: Benign, TOL: Tolerant.

Variant Information		PROVEAN (#SEQ = 108, #CLUSTER = 30)		SIFT (Median Info = 3.01, #SEQ = 49)		Polyphen-2	
POS	Bases altered	SCORE	PREDICTION (cutoff=-2.5)	SCORE	PREDICTION (cutoff=0.05)	Prediction HumVar (PPh2_prob)	Prediction HumDiv (PPh2_prob)
Gly156Ala	1	-5.267	Deleterious	0.05	APF	POD(0.705)	POD(0.908)
Asp185Ala	1	-7.676	Deleterious	0.00	APF	PRD(0.993)	PRD(1.000)
Asp185Lys	2	-6.703	Deleterious	0.00	APF	PRD(0.996)	PRD(1.000)
Trp186Ala	2	-13.438	Deleterious	0.00	APF	PRD(0.991)	PRD(1.000)
Glu187Lys	1	-3.825	Deleterious	0.01	APF	POD(0.875)	PRD(0.986)
Glu187Ala	1	-5.721	Deleterious	0.01	APF	BEN(0.324)	BEN(0.431)
Asp188Lys	2	-3.494	Deleterious	0.04	TOL	POD(0.612)	POD(0.810)
Asp188Ala	1	-4.201	Deleterious	0.07	TOL	BEN(0.375)	POD(0.722)

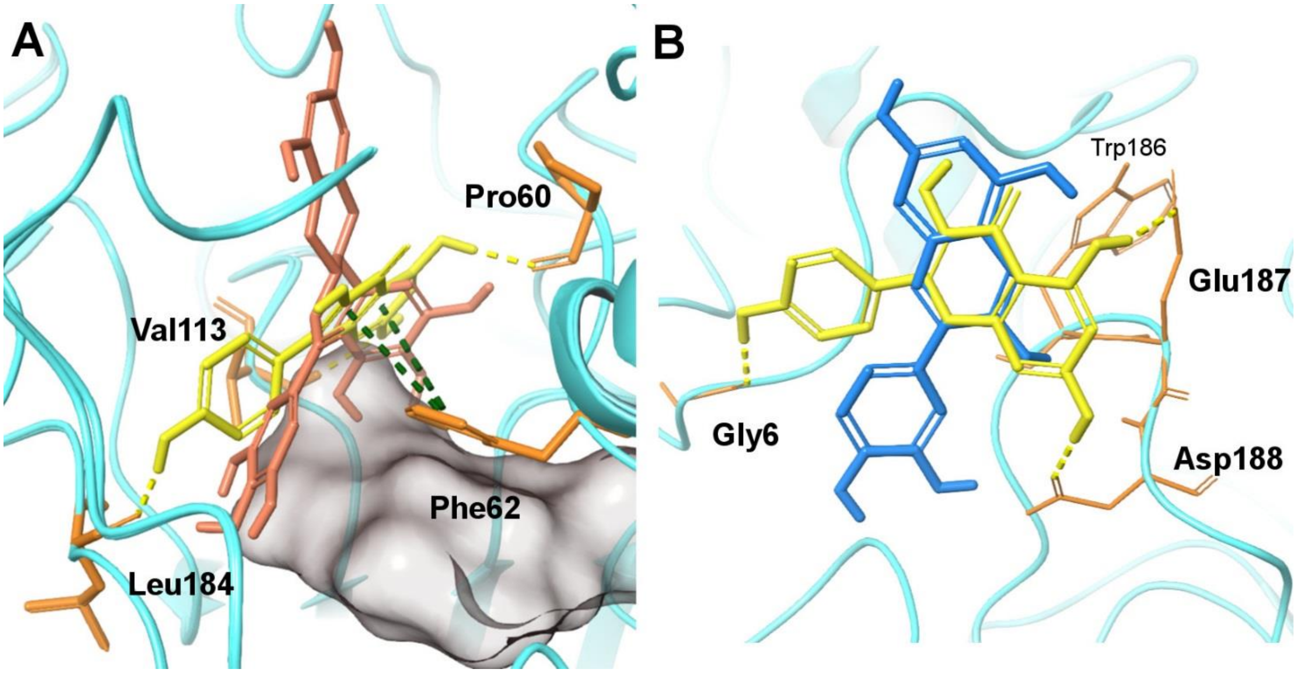

Figure S22. Docking poses of compound **14** (yellow) at SIRT6 inhibitor site (A) together with potent inhibitor compound **5** (brown). Docking pose of compound **14** at putative activator site (B) together with potent activator compound **17** (blue). Yellow dashes indicate hydrogen bonding and green dashes indicate π-π stacking. Grey area represents NAD^+ binding pocket.
